# Associations of lifestyles and frailty status with survival among older adults in China: a nationwide, community-based, prospective cohort study

**DOI:** 10.1186/s12877-025-06878-6

**Published:** 2025-12-19

**Authors:** Haiyan Ruan, Chao Ban, Wei Yi, Liu Yang, Hongli Ma, Liming Zhao, Ziqiong Wang, Kexin Wang, Yi Zheng, Ningying Song, Sen He

**Affiliations:** 1 Department of Cardiology, West China Hospital, Sichuan University, Chengdu, China; 2 Department of Cardiology, Karamay Hospital of Integrated Chinese and Western Medicine, Karamay, China; 3 Department of Cardiology, Hospital of Traditional Chinese Medicine, Shuangliu District, Chengdu, China; 4 Department of Equipment, West China Hospital, Sichuan University, Chengdu, China; 5 Department of Internal Medicine, Hospital of Traditional Chinese Medicine, Maoxian, China; 6 Department of Cardiology, Hospital of Chengdu Office of People’s Government of Tibetan Autonomous Region, Chengdu, China; 7 Department of Otolaryngology‑Head & Neck Surgery, West China Hospital, Sichuan University, Chengdu, China

**Keywords:** China, Frailty status, Lifestyles, Older adults, Overall survival

## Abstract

**Background:**

No studies have examined whether lifestyles mediate the association between frailty status and survival among older adults in China, and research exploring the interactions and joint associations of frailty status and lifestyles on survival is also limited. Therefore, we conducted this study to address these critical gaps in a nationwide, community-based, prospective cohort of older adults in China.

**Methods:**

A total of 17,476 participants (median age: 87.0 [IQR: 80.0–95.0], males: 61.5%) from the Chinese Longitudinal Healthy Longevity Survey conducted between 1998 and 2014 were included, with follow-up until 2018. Frailty index assessed frailty status: robustness, pre-frailty, and frailty. Four lifestyle factors were examined: cigarette smoking, alcohol consumption, physical activity, and diet. The study outcome was overall survival. We performed a mediation analysis of lifestyles on the association between frailty status and survival. Additionally, we assessed the interactions and joint associations of frailty status and lifestyles on survival.

**Results:**

During a median follow-up of 3.4 years, 13,008 deaths (74.4%) were recorded. Compared to robust participants, those with pre-frailty had a 16.0% shorter overall survival (adjusted time ratio [TR]: 0.84, 95% confidence interval [CI]: 0.82–0.86), with lifestyles mediating 11.9% of this difference (95% CI: 9.2%–15.3%); frail participants experienced a 41.0% reduction in survival (adjusted TR: 0.59, 95% CI: 0.57–0.62), with lifestyles mediating 11.1% (95% CI: 9.2%–13.3%). Additionally, more healthy lifestyle factors were associated with longer survival across different frailty levels (p_interaction_=0.090). Furthermore, frail participants with no or one healthy lifestyle factor had a 53.0% shorter survival (adjusted TR: 0.47, 95% CI: 0.44–0.51) compared to robust participants with four healthy lifestyle factors; at the age of 65 years, the former group experienced a reduction in life expectancy of 8.6 years (95% CI: 5.2–12.1) compared to that of the latter group.

**Conclusions:**

Among older adults in China, lifestyles only mediate a small proportion of frailty disparities in overall survival; consequently, without direct interventions for frailty or additional favorable measures, promoting healthy lifestyles alone is insufficient to significantly reduce frailty disparities in survival. Furthermore, individuals of frailty and unhealthy lifestyles experience significantly shorter survival, highlighting the urgent need for targeted interventions for this population.

**Supplementary Information:**

The online version contains supplementary material available at 10.1186/s12877-025-06878-6.

## Background

Frailty is a complex age-related condition characterized by a decline in physiological capacity across multiple organ systems, resulting in an increased susceptibility to stressors [1]. It is a highly prevalent condition that increases with age [2, 3] and a report encompassing 62 countries worldwide indicated the prevalence of frailty among community-dwelling individuals ranged from 11% in those aged 50 to 59 years to 51% in those aged 90 years and older [4]. Frailty has been associated with a broad range of adverse health outcomes, such as disability [5], falls [6], depression [7], dementia [8], cardiovascular disease [9], and notably, mortality [2, 3, 10]; furthermore, frailty also correlates with elevated healthcare expenditures [1, 11]. In the context of global population aging [12], frailty has emerged as a significant health burden worldwide, with substantial implications for both clinical practice and public health; therefore, implementation of targeted strategies is essential to address disparities in frailty-related health.

Lifestyles are often recognized as mediators between frailty status and health, with healthy lifestyles potentially alleviating the excess risk associated with frailty itself. Several studies have investigated the contribution of individual lifestyle factors, as well as their combinations, on the association between frailty status and adverse health outcomes, including depressive symptoms and mortality [13–16]. However, important gaps remain. Firstly, most of these studies predominantly focused on specific cohort populations and were conducted in European countries (the United Kingdom and the Netherlands) [13–15]; therefore, caution is warranted when generalizing these findings to populations from other regions. There are non-negligible differences between Chinese and European populations regarding determinants of health, such as genetics, lifestyles, and hazardous environmental exposures; however, to the best of our knowledge, no studies have investigated the effect of lifestyles on the association between frailty status and survival among older adults in China. Secondly, unhealthy lifestyles also play a significant role in the onset and progression of frailty [2, 17]. Thus, there exists an interplay between frailty status and lifestyles. However, limited research has been conducted on the interactions and joint associations of frailty status and lifestyles on survival.

To fill these crucial research gaps, we aimed to assess whether lifestyles mediate the association between frailty status and overall survival in a nationwide, community-based, prospective cohort of older adults in China. Additionally, we assessed whether adherence to healthy lifestyles was associated with longer overall survival across different levels of frailty status. Lastly, we evaluated the joint association of frailty status and lifestyles with overall survival.

## Methods

### Study participants

The study was based on the Chinese Longitudinal Healthy Longevity Survey (CLHLS), a nationally representative prospective cohort study of older adults, and the general goal of the CLHLS is to shed new light on better understandings of the determinants of healthy aging. The CLHLS collects data regarding the health status, quality of life, and factors contributing to healthy aging among individuals aged 65 and older, with particular emphasis on a substantial portion of the oldest population. The baseline survey of the CLHLS commenced in 1998, with follow-up surveys conducted every two to three years, and data from eight surveys have been made available (i.e., 1998, 2000, 2002, 2005, 2008, 2011, 2014, and 2018). In 1998, the baseline survey, using a multistage, stratified cluster sampling method, was conducted in a randomly selected half of the counties and cities in 22 of 31 provinces in the Chinese mainland, and the population in the survey areas constituted approximately 85.0% of the Chinese population. To mitigate attrition due to mortality and loss to follow-up, new participants have been enrolled since wave 2000, with finally covering 23 of 31 provinces. More details about the CLHLS have been published elsewhere [18], and the data quality of the CLHLS is generally satisfactory when compared to other major aging studies [18, 19]. The CLHLS was approved by Peking University’s research ethics committee (IRB00001052–13074), and conducted in accordance with the principles outlined in the Declaration of Helsinki. Informed consent was given by survey participants prior to their participation.

All variables used in the study were examined since wave 1998, and therefore the study was based on newly recruited participants across waves from 1998 to 2014 (i.e., 1998, 2000, 2002, 2005, 2008, 2011, and 2014), with the final interview conducted in wave 2018. Supplementary Fig. 1 provides a detailed illustration of the participant recruitment process for this study.

### Assessment of frailty status

In the study, frailty status was evaluated using the frailty index, a widely recognized measure of biological age [20]. A 38-item frailty index has been created using data from four waves (i.e., 1998, 2000, 2002, and 2005) of the CLHLS [21], and the index has been widely used [22, 23]. Based on the available data from all waves of the CLHLS, we excluded two variables (i.e., heart rhythm and other chronic diseases) from the original 38-item set; additionally, we removed another variable (i.e., doing housework), as it was considered a domain of physical activity and served as a mediator in this study. Finally, we constructed a modified frailty index comprising 35 variables through a standardized procedure, and the modified index exhibited characteristics similar to those of the general frailty index [24]. Based on the frailty index, frailty status can generally be categorized into five levels [25, 26]: robustness (frailty index ≤ 0.1), pre-frailty (frailty index > 0.1 and ≤ 0.2), mild-frailty (frailty index > 0.2 and ≤ 0.3), moderate-frailty (frailty index > 0.3 and ≤ 0.4), and severe-frailty (frailty index > 0.4). Due to the limited number of participants in the mild-, moderate-, and severe-frailty levels in our study, these were combined, finally resulting in three levels: robustness, pre-frailty, and frailty. For detailed information on assessing frailty status, please refer to Supplementary Method 1.

### Assessment of lifestyle factors and other covariates

Four modifiable lifestyle factors were included in the study, based on previous studies and recommendations from the World Health Organization (WHO): cigarette smoking, alcohol consumption, physical activity, and diet [27–29]. In the present study, never smoking was defined as a healthy level. For alcohol consumption, a healthy level was defined as never or healthy drinking (≤ 15 g of pure alcohol daily, both past and present) [30–32]. Based on the 2020 WHO guideline [33], we defined participants who exercised regularly or engaged in physical activities almost everyday—such as housework, outdoor activities, gardening, and raising domestic animals—as having a healthy level of physical activity. For diet, a total of 12 food items were used to evaluate the healthful diet index for each participant. Intake frequency scores were assigned to each food item: positive scores for healthy foods and reverse scores for less healthy ones; then, the healthful diet index was calculated by summing these scores, with a higher score indicating a more healthful diet. A healthy diet was defined as being in the top two-fifths of this index distribution [29, 34, 35]. For more information on assessing lifestyle factors, please refer to Supplementary Method 2.

Given the interrelated nature of various lifestyle factors and their associations with overall survival, we constructed two types of healthy lifestyle scores: a weighted score and a simple score. These two scoring systems were applied in different analytical contexts. A weighted healthy lifestyle score was created to reflect the varying strengths of associations between different lifestyle factors and overall survival [36, 37]. Moreover, we also constructed a simple healthy lifestyle score [29, 38, 39]. Each lifestyle factor received a score of 1 for a healthy level and 0 for an unhealthy level, finally resulting in a total score from 0 to 4 per participant, with higher scores indicating healthier lifestyles. The weighted score was used for mediation analysis, while the simple score was used to examine the interactions and joint associations of frailty status and lifestyles on overall survival due to its greater explanatory power and public significance in this context. For details on healthy lifestyle scores, please refer to Supplementary Method 2.

Supplementary Table 1 shows detailed information on other available covariates, including sex, age, education, marital status, occupations before the age of 60 years, pension systems, residence, and co-residence.

### Study outcome

The study outcome was overall survival. Survival status and date of death were obtained through interviews with close family members during each survey, and certified from death certificates, hospital admission records, and medical records if available. Follow-up duration was calculated from the time of inclusion in the study to the date of death or to the last follow-up interview for each participant, whichever came first. In the CLHLS dataset, mortality data are generally reliable, except for some recall errors by proxies regarding respondents’ dates of death. In comparison to other major aging studies, the quality of mortality data in the CLHLS is generally satisfactory [18]. 

### Statistical analysis

Supplementary Table 2 presents the distributions of baseline covariates with missing data. In the primay analysis, cases with missing data were excluded under the assumption of missing completely at random. A directed acyclic graph showing the hypothesized association among exposures, mediators, covariates and overall survival is available in Supplementary Method 3. Overall, the analyses comprised five steps: (1) comparing baseline data; (2) assessing whether lifestyles mediate the association between frailty status and overall survival; (3) examining whether adherence to healthy lifestyles is associated with longer overall survival across different levels of frailty status; (4) evaluating the joint association of frailty status and lifestyles with overall survival, while also investigating life expectancy across different groups.

Firstly, the baseline characteristics were presented according to frailty status. Continuous variables were summarized as medians with interquartile ranges (IQR), while categorical variables were reported as counts and percentages. For continuous variables, the *p*-value for trend was calculated using the Pearson test when the row variable followed a normal distribution, and the Spearman test when it exhibited a non-normal distribution. In cases where the row variable was categorical, the *p*-value for trend was derived from the Mantel-Haenszel test of trend.

Secondly, direct standardization was used to calculate age-standardized mortality rates per 1000 person-years, using data from China’s 2020 population census as the reference [40]. Since the proportional hazard assumption was violated, a weibull accelerated failure time model was used instead of a Cox proportional hazard regression model to evaluate the association of frailty status with overall survival (Supplementary Method 3). Accelerated failure time models estimate the time ratio (TR), which reflects the expected event timing in one category relative to a reference group; unlike proportional hazards models, where hazard ratios above 1 indicate higher risk, a TR greater than 1 signifies a longer time to events compared to the reference group. We performed a regression-based causal mediation analysis within the direct counterfactual framework to evaluate how lifestyles mediate the association between frailty status and overall survival [41, 42], and the weighted healthy lifestyle score was used for the mediation analysis (Supplementary Method 3). Standard errors were estimated via bootstrapping with 1000 samples. All models were adjusted for potential confounders: sex, age, education, marital status, occupation prior to the age of 60 years, pension systems, residence, and co-residence.

Thirdly, we examined whether adherence to healthy lifestyles was associated with prolonged survival across different levels of frailty status, using the simple healthy lifestyle score for this analysis. Given that only 599 (3.4%) participants scored 0 points on the simple score, we combined those with 0 and 1 point to enhance statistical power, and the participants were finally categorized into four groups (i.e., 0 or 1, 2, 3, and 4). The reference group was set as participants with a simple healthy lifestyle score of 0 or 1. We performed interaction tests using the likelihood ratio test, which involved comparing models with and without interaction terms.

Lastly, we assessed the joint association of frailty status and lifestyles with overall survival. Participants were categorized into 12 groups based on their frailty status (i.e., robustness, pre-frailty, and frailty) and simple healthy lifestyle score (i.e., 0 or 1, 2, 3, and 4). In the analysis, the reference group was set as participants of robustness and four healthy lifestyle factors. Meanwhile, we calculated the remaining life expectancy across the 12 groups after the specific age of 65 years and before the age of 100 years (Supplementary Method 3). We also assessed the years of life lost among various groups relative to the reference group (i.e., the group of robustness and four healthy lifestyle factors) of the same age at baseline.

To test the robustness of our primary findings, we repeated all analyses stratified by sex (males and females) and age groups (< 80 and ≥ 80 years), along with several sensitivity analyses: (1) to exclude deaths that occurred within the first year of follow-up, thereby reducing potential reverse causation; (2) to address loss to follow-up, we conducted a sensitivity analysis by censoring losses at the median and mean follow-up times; (3) to perform a sensitivity analysis among participants without comorbidities; (4) to mitigate potential bias arising from missing data, we executed mediation analysis following multiple imputation (Supplementary Method 3); (5) to conduct exploratory analyses for cause-specific survival, focusing on cardiovascular disease (CVD) mortality and non-CVD mortality (Supplementary Method 3); (6) to utilize weighted healthy lifestyle score or simple healthy lifestyle score served as a sensitivity analysis under different analytical contexts; (7) to implement several alternative methodologies and indicators in the mediation analysis; and (8) to repeat the primay analysis using the study of osteoporotic fractures index and the 23-item frailty index as indicators of frailty status (Supplementary Method 3).

All analyses were performed with R version 4.2.2 (http://www.R-project.org), with the main packages including “compareGroups”, “survival”, “mice”, “CMAverse”, “lillies”, and “stats”. All tests were two sided, and *p* values < 0.05 were considered statistically significant.

### Role of funding source

The study funder played no role in data collection, data analysis, interpretation of results, manuscript writing, and the decision to submit.

## Results

### Baseline characteristics

Table 1 shows the baseline characteristics of the participants. The study included a total of 17,476 older participants (median age: 87.0 [IQR: 80.0–95.0], males: 61.5%). Participants of frailty were more likely to be women, older, uneducated, unmarried, low occupational grade before the age of 60 years, without pensions, and a higher prevalence of comorbidities. Unhealthy levels of physical activity and diet were more common among participants of frailty; conversely, healthy levels of cigarette smoking and alcohol consumption were more prevalent in this group. Baseline characteristics between included and excluded participants are shown in Supplementary Table 3.

**Table 1:** Baseline characteristics

	All	Robustness	pre-Frailty	Frailty	*p* for trend
No. of participants	17,476	7766	7116	2594	
Sex: male	10,753 (61.5%)	5441 (70.1%)	4105 (57.7%)	1207 (46.5%)	< 0.001
Age (years)	87.0 (80.0–95.0)	83.0 (75.0–91.0)	90.0 (81.0–96.0)	94.0 (87.0–100.0)	< 0.001
Education					< 0.001
No school	9560 (54.7%)	3635 (46.8%)	4192 (58.9%)	1733 (66.8%)	
1 year or more	7916 (45.3%)	4131 (53.2%)	2924 (41.1%)	861 (33.2%)	
Marital status					< 0.001
Not in marriage	11,690 (66.9%)	4492 (57.8%)	5126 (72.0%)	2072 (79.9%)	
In marriage	5786 (33.1%)	3274 (42.2%)	1990 (28.0%)	522 (20.1%)	
Occupation before the age of 60 years					< 0.001
Low occupational grade	12,492 (71.5%)	5335 (68.7%)	5198 (73.0%)	1959 (75.5%)	
Medium occupational grade	3328 (19.0%)	1585 (20.4%)	1293 (18.2%)	450 (17.3%)	
High occupational grade	1656 (9.5%)	846 (10.9%)	625 (8.8%)	185 (7.1%)	
Pension systems					< 0.001
Without pensions	13,777 (78.8%)	5940 (76.5%)	5729 (80.5%)	2108 (81.3%)	
With pensions	3699 (21.2%)	1826 (23.5%)	1387 (19.5%)	486 (18.7%)	
Residence					0.445
Rural	9926 (56.8%)	4365 (56.2%)	4098 (57.6%)	1463 (56.4%)	
Urban	7550 (43.2%)	3401 (43.8%)	3018 (42.4%)	1131 (43.6%)	
Co-residence					0.402
With family members	14,743 (84.4%)	6509 (83.8%)	5979 (84.0%)	2255 (86.9%)	
Alone	2070 (11.8%)	1010 (13.0%)	843 (11.8%)	217 (8.4%)	
In an institution	663 (3.8%)	247 (3.2%)	294 (4.1%)	122 (4.7%)	
Comorbidities					
Hypertension	2634 (15.1%)	553 (7.1%)	1407 (19.8%)	674 (26.0%)	< 0.001
Diabetes	294 (1.7%)	43 (0.6%)	135 (1.9%)	116 (4.5%)	< 0.001
Heart disease	1353 (7.7%)	197 (2.5%)	696 (9.8%)	460 (17.7%)	< 0.001
Stroke or cerebrovascular disease	713 (4.1%)	67 (0.9%)	299 (4.2%)	347 (13.4%)	< 0.001
Bronchitis, emphysema, pneumonia, asthma	2128 (12.2%)	379 (4.9%)	1159 (16.3%)	590 (22.7%)	< 0.001
Tuberculosis	133 (0.8%)	21 (0.3%)	57 (0.8%)	55 (2.1%)	< 0.001
Cancer	72 (0.4%)	3 (< 0.1%)	36 (0.5%)	33 (1.3%)	< 0.001
Gastric or duodenal ulcer	696 (4.0%)	149 (1.9%)	350 (4.9%)	197 (7.6%)	< 0.001
Parkinson’s disease	103 (0.6%)	7 (0.1%)	49 (0.7%)	47 (1.8%)	< 0.001
Bedsore	96 (0.5%)	14 (0.2%)	39 (0.5%)	43 (1.7%)	< 0.001
Cataract	2027 (11.6%)	217 (2.8%)	1025 (14.4%)	785 (30.3%)	< 0.001
Glaucoma	329 (1.9%)	27 (0.3%)	157 (2.2%)	145 (5.6%)	< 0.001
Prostate tumor	658 (3.8%)	97 (1.2%)	326 (4.6%)	235 (9.1%)	< 0.001
Never smoking	9956 (57.0%)	4031 (51.9%)	4221 (59.3%)	1704 (65.7%)	< 0.001
Never or healthy drinking	12,255 (70.1%)	5207 (67.0%)	5088 (71.5%)	1960 (75.6%)	< 0.001
Healthy physical activity	11,392 (65.2%)	6301 (81.1%)	4402 (61.9%)	689 (26.6%)	< 0.001
Healthy diet	7643 (43.7%)	3751 (48.3%)	2943 (41.4%)	949 (36.6%)	< 0.001
HLS	2.0 (2.0–3.0)	3.0 (2.0–3.0)	2.0 (2.0–3.0)	2.0 (1.0–3.0)	< 0.001
Weighted HLS	0.58 (0.36–0.86)	0.72 (0.50–0.86)	0.58 (0.36–0.86)	0.36 (0.28–0.50)	< 0.001
Frailty index	0.11 (0.08–0.16)	0.07 (0.05–0.09)	0.14 (0.12–0.16)	0.26 (0.23–0.32)	< 0.001

Values are median (IQR) or *n* (%)

*Abbreviations*: *HLS *healthy lifestyle score, *IQR *interquartile range

### Mediation analysis of lifestyles on associations between frailty status and overall survival

During a follow-up period of 82156.3 person-years (median: 3.4 years, IQR: 1.8–6.5 years), a total of 13,008 deaths (74.4%) were recorded. After adjustment for potential confounders, greater frailty severity was associated with shorter overall survival, and healthy lifestyles were associated with longer overall survival (Supplementary Tables 4 and 5 and Supplementary Fig. 2). In addition, there was an inverse correlation between frailty severity and healthy lifestyles (Supplementary Tables 6 and Supplementary Fig. 3).

Table 2 shows the results of mediation analysis. Compared with participants of robustness, those of pre-frailty had a 16.0% shorter overall survival (adjusted TR: 0.84, 95% CI: 0.82–0.86), with lifestyles (weighted healthy lifestyle score) accounting for 11.9% of this disparity (95% CI: 9.2%–15.3%). In comparison to participants of robustness, those of frailty experienced a significant reduction of 41.0% in overall survival (adjusted TR: 0.59, 95% CI: 0.57–0.62), with lifestyles mediating 11.1% of the difference (95% CI: 9.2%–13.3%). The results of all sensitivity analyses were largely consistent (Supplementary Tables 7–12); however, it was observed that the mediated proportion decreased when the weighted healthy lifestyle score was replaced with the simple healthy lifestyle score (Supplementary Table 11) and in the subgroup of participants aged less than 80 years (Supplementary Table 7).

**Table 2:** Mediation analysis of lifestyles on the association between frailty status and overall survival

	Robustness	pre-Frailty	Frailty
No. of participants	7766	7116	2594
No. of deaths	5164	5528	2316
Total person-years of follow-up	45370.9	29903.5	6881.9
Mortality rate (95% CI)^a^, per 1000 person-years	67.3 (64.7–69.9)	82.4 (78.0–87.0)	131.7 (116.2–149.3)
Association^b^			
Total effect; TR (95% CI)	1.00 (ref)	0.84 (0.82–0.86)	0.59 (0.57–0.62)
Natural direct effect; TR (95% CI)		0.86 (0.83–0.88)	0.64 (0.61–0.66)
Natural indirect effect; TR (95% CI)		0.978 (0.973–0.982)	0.929 (0.918–0.938)
Mediation proportion; % (95% CI)		11.9 (9.2–15.3)	11.1 (9.2–13.3)

*Abbreviations*: *CI* confidence interval, *TR* time ratio

^a^Age-standardized mortality rates, with data from the 2020 population census in China as the reference

^b^Natural direct effect and natural indirect effect estimated the effect of frailty status on overall survival that did not or did act through the mediator (i.e., lifestyles, measured by weighted healthy lifestyle score), respectively. The mediation proportion estimated the percentage of the effect of frailty status, on the log (TR) scale, that acted through the mediator (i.e., lifestyles, measured by weighted healthy lifestyle score). The model was adjusted for sex, age, education, marital status, occupation prior to the age of 60 years, pension systems, residence, and co-residence. When examining exposure-mediator interaction, the overall proportion attributable to interaction was statistically insignificant; therefore, the present result was calculated without accounting for exposure-mediator interaction

Regarding each lifestyle factor, both physical activity and diet partially mediated the association between frailty status and overall survival. Specifically, physical activity accounted for a mediation effect ranging from 8.9% to 9.7%, while diet contributed to this association with mediation proportions varying from 0.5% to 1.3%. However, neither cigarette smoking nor alcohol consumption mediated this association (Supplementary Table 13).

### Associations of healthy lifestyles with overall survival across different levels of frailty status

**Fig. 1:**
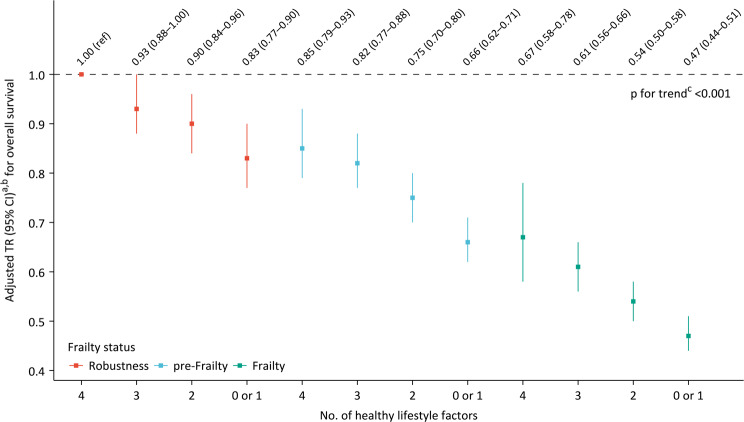
Associations of lifestyles with overall survival by different levels of frailty status. Note: ^a^ Age-standardised mortality rates, with data from the 2020 population census in China as the reference. ^b^ With adjustment for sex, age, education, marital status, occupation prior to the age of 60 years, pension systems, residence, and co-residence. ^c^ The *p*-value for trend was derived from Wald tests assessing the linear association between the number of healthy lifestyle factors, quantified as a numerical value (1–4), and the risk of overall survival. Abbreviations: CI=confidence interval, TR=time ratio

Among participants with different levels of frailty status, more healthy lifestyle factors were associated with longer overall survival (all *p*-values for trend < 0.001), and no significant interaction was found between lifestyles and frailty status on overall survival (*p*-value for interaction = 0.090, Fig. 1). Overall, the subgroup and sensitivity analyses produced consistent findings (Supplementary Figs. 4–6). However, a significant interaction was observed among participants aged 80 years and older (*p*-value for interaction = 0.002), where healthy lifestyles notably extended overall survival among participants of frailty compared to those of robustness (Supplementary Fig. 4). Furthermore, the *p*-values for trend were not statistically significant among pre-frail participants under the age of 80, frail participants without comorbidities, and robust participants in assessing CVD-specific survival (Supplementary Figs. 4–6); nevertheless, point estimates for overall survival consistently favored an increased number of healthy lifestyle factors.

### Joint associations of frailty status and lifestyles on overall survival

**Fig. 2:**
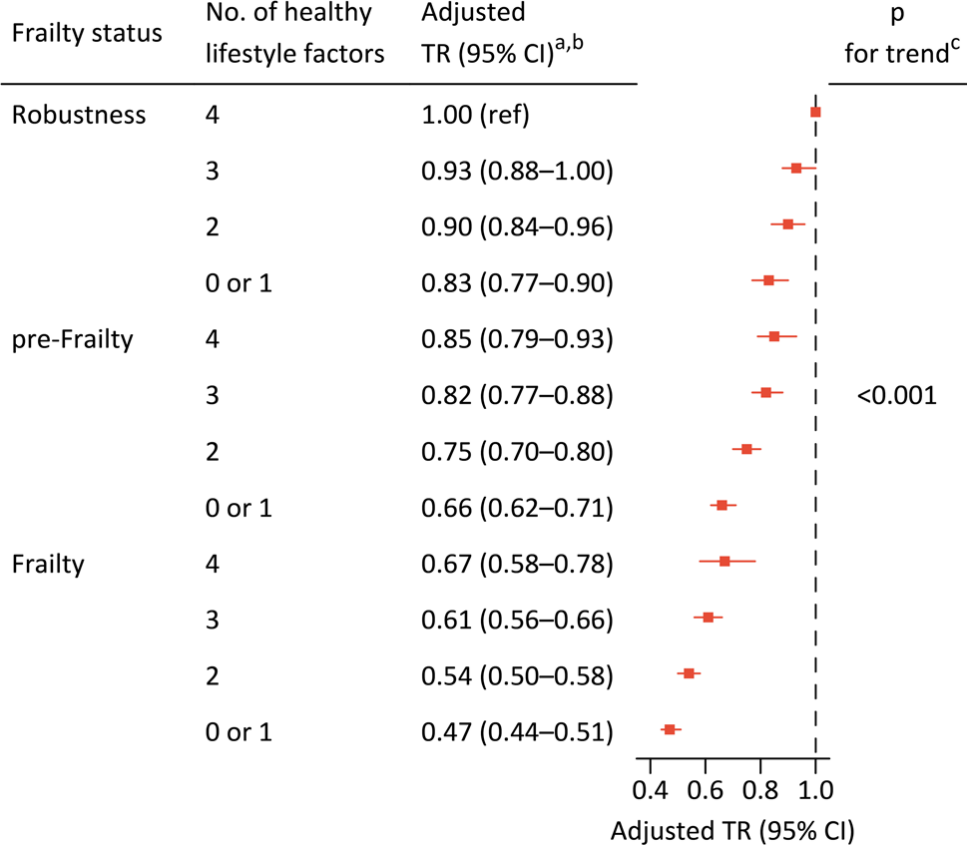
Joint associations of frailty status and lifestyles with overall survival. Note: ^a^ With adjustment for sex, age, education, marital status, occupation prior to the age of 60 years, pension systems, residence, and co-residence. ^b^ Age-standardised mortality rates for each group are shown in Fig. 1. ^c^ The *p*-value for trend was derived from Wald tests assessing the linear association between a combination of frailty status and the number of healthy lifestyle factors, represented as a numerical value (1–12), and the risk of overall survival. Abbreviations: CI=confidence interval, TR=time ratio

Figure 2 shows the joint association of frailty status and lifestyles on overall survival. In general, the standardized mortality rate increased with greater frailty severity and fewer healthy lifestyle factors. For participants of frailty and no or one healthy lifestyle factor, the mortality rate was 165.5 per 1000 person-years (95% CI: 132.7–207.0), nearly three times higher than those of robustness and four healthy lifestyle factors (58.2 per 1000 person-years [95% CI: 52.4–64.6]). Additionally, greater frailty severity coupled with fewer healthy lifestyle factors was significantly associated with shorter overall survival. The TR for participants of frailty and no or one healthy lifestyle factor compared with those of robustness and four healthy lifestyle factors were 0.47 (95% CI: 0.44–0.51) for overall survival (Fig. 2). These results remained consistent across all subgroup and sensitivity analyses (Supplementary Figs. 7–9).

**Fig. 3:**
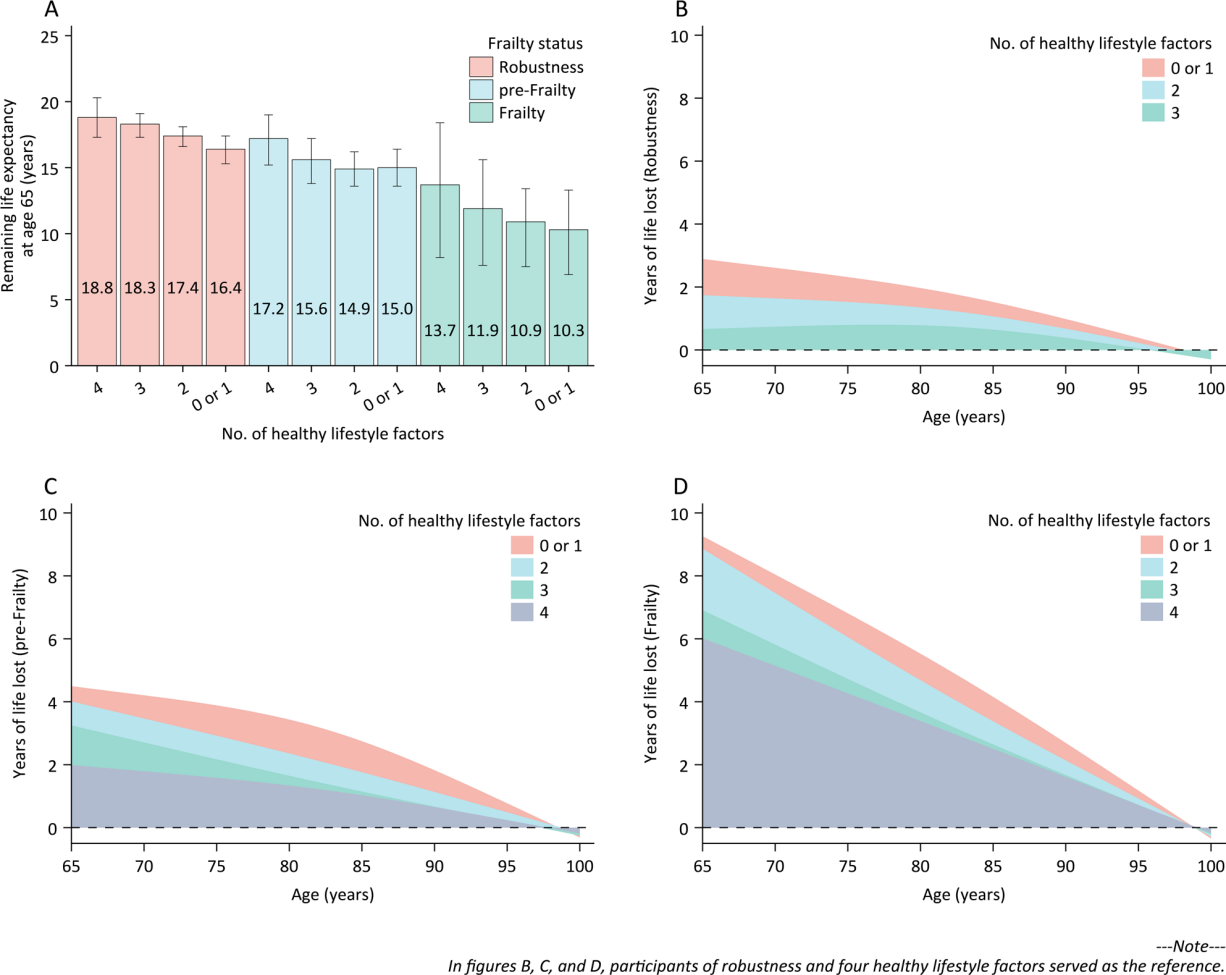
Life expectancy and years of life lost by frailty status and number of healthy lifestyle factors. **A** Remaining life expectancy at the age of 65 years by a combination of frailty status and the number of healthy lifestyle factors. **B**–**D** Age-specific years of life lost among various groups in comparison to participants of robustness and four healthy lifestyle factors

The remaining life expectancy at the age of 65 years decreased with increased frailty severity, and this trend was similarly observed in relation to a reduced number of healthy lifestyle factors (Supplementary Fig. 10). Moreover, the joint association of frailty status and lifestyles on life expectancy at the age of 65 years showed that remaining life expectancy significantly decreased with greater frailty severity coupled with fewer healthy lifestyle factors. The remaining life expectancy at the age of 65 was 18.8 years (95% CI: 17.3–20.3) for participants of robustness and four healthy lifestyle factors, while those of frailty and no or one healthy lifestyle factor had a life expectancy of 10.3 years (95% CI: 6.9–13.3) (Fig. 3A). At the age of 65 years, participants in different groups experienced a loss of life expectancy ranging from 0.6 years (95% CI: -1.2 to 2.1; robustness and three healthy lifestyle factors) to 8.6 years (95% CI: 5.2–12.1; frailty and no or one healthy lifestyle factor) compared to participants of robustness and four healthy lifestyle factors (Fig. 3B–D). In exploratory analyses of cause-specific survival, participants of frailty and no or one healthy lifestyle factor lost an average of 4.0 years of life after the age of 65 years compared to participants of robustness and four healthy lifestyle factors. This loss was attributed to increased deaths from CVD (1.7 years, 42.5% of total loss) and non-CVD causes (2.3 years, 57.5% of total loss) (Supplementary Fig. 11).

## Discussion

The present study is the first to explore the association between frailty status, lifestyles, and overall survival in a nationwide, community-based, prospective cohort of older adults in China. There were three key findings. Firstly, greater frailty severity was associated with shorter overall survival, but only 11.1% to 11.9% of the associations were mediated by lifestyles. Secondly, more healthy lifestyle factors were associated with longer overall survival across different levels of frailty status. Thirdly, greater frailty severity combined with fewer healthy lifestyle factors was significantly associated with shorter overall survival. Participants of frailty and no or one healthy lifestyle factor experienced the shortest overall survival—53.0% less than those of robustness and four healthy lifestyle factors; at the age of 65 years, the former group experienced a reduction in life expectancy of 8.6 years (95% CI: 5.2–12.1) compared to that of the latter group.

Frailty disparities in mortality have been widely discussed among older adults [1, 3, 25, 43, 44]. Analysis of the UK Biobank data showed that frailty significantly increased the risk of all-cause mortality compared to robustness among older adults (hazard ratio [HR]: 2.42 [95% CI: 2.09–2.80] for men; HR: 2.53 [95% CI: 2.10–3.04] for women) [44]. A study using data from the China Kadoorie Biobank further corroborated this association, revealing frailty was associated with an elevated risk of all-cause mortality among older adults (HR: 2.62, 95% CI: 2.50–2.74), as well as cause-specific mortality, with HRs ranging from 1.27 (95% CI: 1.15–1.40, cancer) to 5.60 (95% CI: 4.96–6.33, respiratory disease) [3]. Likewise, a meta-analysis of 11 prospective studies involving 35,538 older adults found the risk of mortality in the frail group was higher than that in the robust group (summary HR: 2.00, 95% CI: 1.73–2.32) [43]. Our analysis confirmed the existence of disparities in mortality related to frailty among older adults; therefore, it is essential to explore potential methods for reducing these disparities.

Lifestyles are frequently regarded as mediators between risk factors and health outcomes [29, 45–49], and the reduction of mortality by adhering to a healthy lifestyle has been well-reported [36, 39, 50]. Recently, several studies have investigated the contribution of lifestyles on the association between frailty status and mortality. Based on a large nationwide cohort comprising 411,987 adults (mean age: 56.3 years) from the United Kingdom [14], the findings indicated overall lifestyle—assessed through smoking status, alcohol intake, physical activity, diet scores, and body mass index—partially mediated the association between frailty and total respiratory disease mortality (mediated proportion: 5.1% [95% CI: 4.4%–5.9%]), and the mediated proportion of lifestyles on pre-frailty mortality was 5.1% (95% CI: 3.3%–7.9%). Another study involving 91,906 British adults aged 60 and older suggested overall lifestyle—evaluated based on physical activity, diet, alcohol intake, and smoking status—partially mediated the association between frailty and all-cause mortality (mediated proportion: 13.6% [95% CI: 11.3%–16.2%]), and the mediated proportion of lifestyles on pre-frailty mortality was 4.3% (95% CI: 3.2%–5.5%) [15]. Our findings were consistent with previous studies and further elaborated that overall lifestyle partially mediated the association between frailty status and overall survival among older adults in China; however, the current evidence indicating a low mediation proportion suggests that promoting healthy lifestyles alone will not significantly reduce frailty disparities in mortality without direct interventions for frailty or additional supportive measures. Moreover, our results, along with previous studies [13, 15], suggested that not all individual lifestyle factors mediated the association between frailty status and mortality, with physical activity likely being the most significant mediator [15]. A previous study suggested physical activity had the highest mediated proportion (7.7% [95% CI: 5.0–10.4]) on the association between frailty status and mortality among the four lifestyle factors examined, while alcohol intake did not mediate the association [15]. Another investigation involving 1477 Dutch individuals aged 65 and older found two lifestyle factors—alcohol use and sleep problems—did not serve as mediators in the association between frailty status and mortality [13]. 

Despite the fact that lifestyles mediated only a small portion of the disparities in frailty-related overall survival, more healthy lifestyle factors were found to be associated with longer overall survival across different levels of frailty status among older adults in China, supporting an important role of healthy lifestyles in reducing death burden. Although significant interactions were not observed, the protective association between healthy lifestyles and overall survival may be more pronounced among individuals with frailty, particularly those aged 80 and above (*p*-value for interaction = 0.002). These findings emphasize the importance of lifestyle modification, especially for individuals of frailty, and those may be more beneficial from healthy lifestyles, in line with the vulnerability hypothesis [51]. A previous study from the United Kingdom also found more healthy lifestyle factors were associated with a lower risk of total respiratory disease mortality, regardless of frailty levels; however, no differences were noted across varying levels of frailty status [14]. The exact reasons for the inconsistent findings remained unclear, possibly due to varying definitions of frailty, lifestyle factors, and population characteristics.

In the study, we found that greater frailty severity combined with fewer healthy lifestyle factors was significantly associated with shorter overall survival, aligning with previous research on the effects of frailty status and lifestyles on respiratory disease mortality [14]. In addition, several studies have indicated that frailty can adversely affect life expectancy [52, 53]. Our work corroborates these findings, and in this study, we further found greater frailty severity combined with fewer healthy lifestyle factors was significantly associated with reduced remaining life expectancy among older adults in China. These findings indicate that individuals of frailty and unhealthy lifestyles bear a substantial death burden, highlighting the urgent necessity for targeted interventions aimed at this population. However, considering the dynamic nature of frailty status [54] and the modifiable aspects of lifestyle factors, these results also suggest substantial potential to enhance life expectancy in China through initiatives focused on promoting healthy lifestyles and addressing frailty. Recently, the Chinese government has made considerable efforts to realize the potential, including the development of localized tools and guidelines for frailty assessment [55], the expansion of geriatric medicine departments [55], and the implementation of population-wide healthy lifestyle interventions [56, 57]. 

Major strengths of this study include the large sample size, consistent findings from sensitivity and subgroup analyses, and the completeness of data provided by the CLHLS, which allowed us to systematically explore the complex association of lifestyles and frailty status with overall survival among older adults in China. Nevertheless, several limitations should be acknowledged. Firstly, the information regarding the frailty index and lifestyles was primarily self-reported and measured only once, which inevitably introduced measurement errors. Secondly, we were unable to capture long-term frailty trajectories as well as changes in lifestyles over time; thus, future studies employing repeated measurements are recommended. Thirdly, although we controlled for key personal characteristics, residual confounding may still exist, and causal inference cannot be established due to the inherent nature of observational studies. Fourthly, we developed two types of lifestyle scores tailored for different analytical contexts. While a weighted lifestyle score was created, it does not fully account for the complex interactions among various lifestyle factors; moreover, the weights assigned were specific to this study. Additionally, deriving a lifestyle score from a simple sum of healthy lifestyle factors assumes that all such factors exert equal effects on health outcomes—a premise that may not hold true. Fifthly, individuals excluded from the analysis due to missing covariates were more likely to be frail; consequently, our findings may underestimate frailty inequity in overall survival. However, results remained consistent even after imputing missing covariates. Lastly, the participants in this study were predominantly very old (median age: 87.0 years), which may limit the generalizability of our findings to younger older adults. This advanced age may also explain the modest mediation effect observed for lifestyles; at this stage of life, the cumulative burden of physiological decline and comorbidities could overshadow the influence of modifiable lifestyles. However, several factors enhance our confidence in the robustness of our findings for the broader older adult population. Internally, a sensitivity analysis stratified by age (< 80 and ≥ 80 years) yielded consistent results (Supplementary Table 7). In addition, similar conclusions were reported in a UK Biobank study involving 91,906 individuals aged ≥ 60 years (mean age: 64) [15]. Nevertheless, caution is warranted when extrapolating these results to significantly younger populations of older adults.

## Conclusion

Among older adults in China, greater frailty severity was found to be associated with shorter overall survival, and only a minor portion of the association was mediated by lifestyles; therefore, promoting healthy lifestyles alone may not significantly reduce frailty disparities in overall survival without direct interventions for frailty or additional favorable measures. Nevertheless, more healthy lifestyle factors were associated with longer overall survival across different levels of frailty status, underscoring the role of healthy lifestyles in reducing death burden. Morerover, individuals of frailty and unhealthy lifestyles experienced a significant reduction in overall survival, highlighting the critical need for targeted interventions for this population, which is not only beneficial for achieving the life expectancy goals outlined in the Healthy China Action (2019–2030) [58] but also contributes to the broader societal objective of promoting healthy aging.

## Supplementary Information

Supplementary Material 1.

## Data Availability

All CLHLS data are publicly available and can be downloaded from the following websites: (1) https://opendata.pku.edu.cn/dataverse/CHADS; (2) [https://www.icpsr.umich.edu/icpsrweb/NACDA/series/487].

## Abbreviations

CLHLS: the Chinese Longitudinal Healthy Longevity Survey
WHO: the World Health Organization
IQR: interquartile ranges
CI: confidence interval
TR: time ratio
CVD: cardiovascular disease
HLS: healthy lifestyle score
