# Supplementary material for: Associations of lifestyles and frailty status with survival among older adults in China: a nationwide, community-based, prospective cohort study
Source: BMC Geriatr. 2025 Dec 19;26:95. doi: 10.1186/s12877-025-06878-6 (PMC12831328; doi:10.1186/s12877-025-06878-6)
Supplement: Supplementary file 1 — Supplementary Material 1. [file 12877_2025_6878_MOESM1_ESM.pdf]

Associations of lifestyles and frailty status with survival among older adults in China: a nationwide, community-based, prospective cohort study

Haiyan Ruan[1,3], Chao Ban[4], Wei Yi[5], Liu Yang[2], Hongli Ma[2], Liming Zhao[6], Ziqiong Wang[1], Kexin Wang[1], Yi Zheng[1], Ningying Song[7]*, Sen He[1,2*]
(Haiyan Ruan and Chao Ban equally contributed to the article)

1. Department of Cardiology, West China Hospital, Sichuan University, Chengdu, China.
2. Department of Cardiology, Karamay Hospital of Integrated Chinese and Western Medicine, Karamay, China.
3. Department of Cardiology, Hospital of Traditional Chinese Medicine, Shuangliu District, Chengdu, China.
4. Department of Equipment, West China Hospital, Sichuan University, Chengdu, China.
5. Department of Internal Medicine, Hospital of Traditional Chinese Medicine, Maoxian, China.
6. Department of Cardiology, Hospital of Chengdu Office of People's Government of Tibetan Autonomous Region, Chengdu, China
7. Department of Otolaryngology-Head & Neck Surgery, West China Hospital, Sichuan University, Chengdu, China.

*Corresponding author:

Sen He: Department of Cardiology, West China Hospital, Sichuan University, Chengdu, China; E-mail: hesensubmit@163.com (or: hesen_sky@scu.edu.cn).

Ningying Song: Department of Otolaryngology-Head & Neck Surgery, West China Hospital, Sichuan University, Chengdu, China; E-mail: songningying2@163.com

This study was supported by the Sichuan Science and Technology Program, China (Grant No. 2022YFS0186), the National Natural Science Foundation of China (Grant No. 81600299), the Science and Technology Major Project of Tibetan Autonomous Region of China (Grant No. XZ202201ZD0001G01), and the Key Research and Development Projects of Chengdu Science and Technology Bureau (Grant No. 2022-YF05-01335-SN).

List of supplementary materials

Supplementary Method 1: assessment of frailty status 3
Supplementary Method 2: assessment of lifestyle factors, construction of healthy lifestyle score 5
1. Assessment of lifestyle factors 5
2. Construction of healthy lifestyle score: weighted and simple methods 7
Supplementary Method 3: statistical analysis 8
1. Directed acyclic graphs 8
2. Accelerated failure time models 8
3. Mediation analysis 8
4. Remaining life expectancy and years of life lost 9
5. Multiple imputation 9
6. Cause-specific survival 9
7. Study of osteoporotic fractures index 9
8. A 23-item frailty index 9
Supplementary Table 1. Definitions of baseline covariates in the present study 12
Supplementary Table 2. Distributions of baseline covariates with missing data 14
Supplementary Table 3. Baseline characteristics of participants included or excluded from analyses 15
Supplementary Table 4. Associations between frailty status and overall survival 16
Supplementary Table 5. Associations between healthy lifestyle score, as well as each healthy lifestyle factor, and overall survival 17
Supplementary Table 6. Linear regression for the association between frailty index and healthy lifestyle score 17
Supplementary Table 7. Mediation analysis of lifestyles on the association between frailty status and overall survival: subgroup analysis 18
Supplementary Table 8. Mediation analysis of lifestyles on the association between frailty status and overall survival: reverse causation, censoring losses at different time points, and without comorbidities 19
Supplementary Table 9. Mediation analysis of lifestyles on the association between frailty status and survival: multiple imputation and cause-specific survival 20
Supplementary Table 10. Mediation analysis of lifestyles on the association between frailty status and overall survival: several additional methodologies for mediation analysis (n=17476) 20
Supplementary Table 11. Mediation analysis of lifestyles on the association between frailty status and overall survival: alternative methodologies used for the mediator and the frailty status (n=17476) 21
Supplementary Table 12. Mediation analysis of lifestyles on the association between frailty status and overall survival: using the study of osteoporotic fractures index or the 23-item frailty index to assess frailty status 21
Supplementary Table 13. Mediation analysis of lifestyles on the association between frailty status and overall survival: each lifestyle factor (n=17476) 22
Supplementary Figure 1. Flow chart of participants selection for main analyses 23
Supplementary Figure 2. Associations between frailty index, weighted healthy lifestyle score, and overall survival 23
Supplementary Figure 3. Correlations between frailty index and weighted healthy lifestyle score 24
Supplementary Figure 4. Associations of lifestyles with overall survival by frailty status: subgroup analysis 25
Supplementary Figure 5. Associations of lifestyles with overall survival by frailty status: reverse causation, censoring losses at different time points, without comorbidities, and multiple imputation 26
Supplementary Figure 6. Associations of lifestyles with overall survival by frailty status: weighted healthy lifestyle score, cause-specific survival, SOF index, and 23-item frailty index 27
Supplementary Figure 7. Joint associations of frailty status and lifestyles with overall survival: subgroup analysis 28
Supplementary Figure 8. Joint associations of frailty status and lifestyles with overall survival: reverse causation, censoring losses at different time points, without comorbidities, and multiple imputation 29
Supplementary Figure 9. Joint associations of frailty status and lifestyles with overall survival: weighted healthy lifestyle score, cause-specific survival, SOF index, and 23-item frailty index 30
Supplementary Figure 10. Estimated remaining life expectancy at the age of 65 years 31
Supplementary Figure 11. Attribution of the causes of death 31
Supplementary file: STROBE checklist—all the items listed below have been confirmed, and item 6(b) is not relevant to the present study 32

Supplementary Method 1: assessment of frailty status

In the study, frailty status was assessed by the frailty index, which is one of the extensively used measures of biological age.[1] The frailty index, derived from the accumulation of deficits approach, condenses various health variables into a single continuous score that reflects an individual's overall health, and a reliable frailty index typically requires at least 30 variables across multiple domains.[2] The responses for each variable must be recoded to a scale from 0 to 1, where 0 indicates no deficit (the healthiest state) and 1 indicates full deficit (the unhealthiest state). Dichotomous variables are coded 0 and 1 (e.g., no hyptertension =0, hyptertension =1). Interval or ordinal variables with three response levels are coded 0, 0.5, and 1, and those with four levels are coded 0, 0.33, 0.67, and 1. A similar methodology is applied for variables with more than four response levels. Finally, the frailty index can be calculated by dividing the sum of recoded values (i.e., the sum of deficits) by the number of valid variables for a specific individual. For instance, in a dataset with 50 variables, an individual with a deficit sum of 2 and 45 valid variables (i.e., five variables with missing values) would have a frailty index of 0.04 (2/45). In the same dataset, an individual with a deficit sum of 5.5 and no variables with missing values will have an index of 0.11 (5.5/50). The frailty index is a continuous variable that ranges from 0.00 to 1.00, with higher values indicating a greater degree of frailty. Importantly, the frailty index should not be calculated for individuals if more than 20% of total variables are missing (e.g., over ten variables with missing values in a dataset with 50 variables).[2] In this study, to rigorously eliminate potential biases (i.e., those caused by certain variables with missing values that may be associated with either smaller or larger deficits), each participant was ensured to have a complete set of frailty index variables.

A 38-item frailty index has been created using data from four waves (i.e., 1998, 2000, 2002, and 2005) of the Chinese Longitudinal Healthy Longevity Survey (CLHLS),[3] and the index has been widely used.[4,5] Based on the available data from all waves of the CLHLS, we excluded two variables (i.e., heart rhythm and other chronic diseases) from the original 38-item set; additionally, we removed another variable (i.e., doing housework), as it is considered a domain of physical activity and served as a mediator in this study. Finally, we constructed a modified frailty index with 35 variables using a standardized procedure,[2] and detailed information about these variables is provided in the table below.

Generally, the frailty index constructed using population data shares certain characteristics:[2] a right-skewed frequency distribution, index less than 0.70 for at least 99% of the samples, higher mean frailty index in females than males, and a positive correlation with age. In our study, the frailty index demonstrated similar characteristics (as shown in Figures A and B below).

Based on the frailty index, frailty status can generally be categorized into five levels:[6,7] robustness (frailty index ≤0.1), pre-frailty (frailty index >0.1 and ≤0.2), mild-frailty (frailty index >0.2 and ≤0.3), moderate-frailty (frailty index >0.3 and ≤0.4), and severe-frailty (frailty index >0.4). Due to the limited number of participants in the mild-, moderate-, and severe-frailty levels in our study (as shown in Figure A below), these were combined, finally resulting in three levels: robustness, pre-frailty, and frailty.

Table. Variables used to construct frailty index

	Variables	Data type	Corresponding scores based on the response options[a]
1	Self-reported health	Ordinal	very good =0, good =0.25, so so =0.5, bad =0.75, very bad =1
2	Feel fearful or anxious	Ordinal	always =1, often =0.75, sometimes =0.5, seldom =0.25, never =0
3	Feel useless with age	Ordinal	always =1, often =0.75, sometimes =0.5, seldom =0.25, never =0
4	Look on the bright side of things	Ordinal	always =0, often =0.25, sometimes =0.5, seldom =0.75, never =1
5	Make own decisions	Ordinal	always =0, often =0.25, sometimes =0.5, seldom =0.75, never =1
6	Keep my belongings neat and clean	Ordinal	always =0, often =0.25, sometimes =0.5, seldom =0.75, never =1
7	ADLs: bathing	Ordinal	without assistance =0, one part assistance =0.5, more than one part assistance =1
8	ADLs: dressing	Ordinal	without assistance =0, need assistance for trying shoes =0.5, assistance in getting clothes and getting dressed =1
9	ADLs: toileting	Ordinal	without assistance =0, assistance in cleaning or arranging clothes =0.5, don't use toilet =1
10	ADLs: transferring	Ordinal	without assistance =0, with assistance =0.5, bedridden =1
11	ADLs: continence	Ordinal	without assistance =0, occasional accidents =0.5, incontinent =1
12	ADLs: feeding	Ordinal	without assistance =0, with some help =0.5, need feeding = 1
13	Functional limitations: hand behind neck	Ordinal	both hands =0, left hand =0.5, right hand =0.5, neither hand =1
14	Functional limitations: hand behind lower back	Ordinal	both hands =0, left hand =0.5, right hand =0.5, neither hand =1
15	Functional limitations: able to stand up from sitting	Ordinal	yes, without using hands =0; yes, using hands =0.5; no =1
16	Functional limitations: able to pick up a book from the floor	Ordinal	yes, standing =0; yes, sitting =0.5; no =1
17	Functional limitations: able to use chopsticks to eat	Binary	yes =0, no =1
18	Visual function	Ordinal	can see and distinguish =0, can see only =0.5, cannot see =1, blind =1
19	Able to hear	Binary	yes (with or without hearing aids) =0, no (no; partly, despite using hearing aids) =1

	Variables	Data type	Corresponding scores based on the response options[a]
20	Number of steps used to turn around a 360 degree turn without help	Binary	<6 steps =0,<br>≥6 steps or cannot turn around =1
21	Number of times suffering from serious illness in the past two years	Binary	no serious illness =0, suffering from serious illness in the past two years ≥1 time(s) or bedridden all the year around =1
22	Interviewer rated health	Ordinal	surprisingly healthy =0, relatively healthy =0, moderately ill =0.5, very ill =1
23	Hypertension	Binary	yes =1, no =0
24	Diabetes	Binary	yes =1, no =0
25	Heart disease	Binary	yes =1, no =0
26	Stroke or cerebrovascular disease	Binary	yes =1, no =0
27	Bronchitis, emphysema, pneumonia, asthma	Binary	yes =1, no =0
28	Tuberculosis	Binary	yes =1, no =0
29	Cancer	Binary	yes =1, no =0
30	Gastric or duodenal ulcer	Binary	yes =1, no =0
31	Parkinson's disease	Binary	yes =1, no =0
32	Bedsore	Binary	yes =1, no =0
33	Cataract	Binary	yes =1, no =0
34	Glaucoma	Binary	yes =1, no =0
35	Prostate tumor	Binary	yes =1, no =0

[a] If response options are labeled as 'don't know' or 'missing' in the codebook, both were treated as 'missing' and excluded from the construction of the frailty index.

Abbreviations: ADLs=activities of daily living.

Figure. Characteristics of frailty index

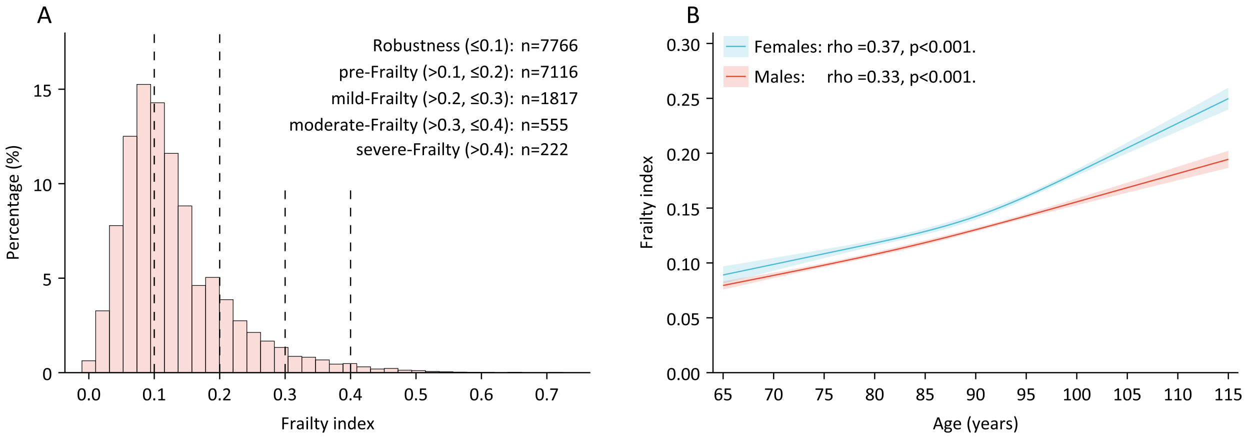

Note:

(A) Frailty index for all participants: median=0.11, mean=0.13, range=0.00–0.72; a right-skewed frequency distribution; the 99th percentile index =0.42 (<0.70).

(B) Frailty index by sex and age: higher mean frailty index in females than males (0.15±0.09 vs. 0.12±0.08, p<0.001); a positive correlation between frailty index and age, with Spearman correlations of 0.37 for females and 0.33 for males, respectively. The curves were generated by a linear regression model with a restricted cubic spline with three knots at the 10th, 50th, and 90th percentiles.

Supplementary Method 2: assessment of lifestyle factors, construction of healthy lifestyle score

1. Assessment of lifestyle factors

Four modifiable lifestyle factors were included in the study, based on previous studies and recommendations from the World Health Organization (WHO): cigarette smoking, alcohol consumption, physical activity, and diet.[8-10] All lifestyle factors were obtained through structured questionnaires. The assessment of each lifestyle factor is detailed as follows.

1.1 Cigarette smoking

In the CLHLS, cigarette smoking is assessed through the following five self-report questions:

(1) Do you smoke at present (response: yes/no)?
(2) Did you smoke in the past (response: yes/no)?
(3) How old were you when you began smoking (response: age)?
(4) How old were you when you stopped smoking if you don`t smoke at present (response: age)?
(5) If you smoke at the present time (or smoked in the past), how many times per day on average do (or did) you smoke (response: times)?

On the whole, cigarette smoking was classified as current, former, or never. Although a pooled analysis indicated that the risk of all-cause mortality among former smokers approached that of never smokers after 15 years of smoking cessation, the overall mortality risk remained high, despite lacking statistical significance.[11] In addition, several other studies have also shown that that both current and former smokers exhibit a higher mortality risk in comparison to individuals who have never smoked.[12,13] Moreover, despite the CLHLS questionnaire does not inquire about the reasons for smoking cessation, there exists a possibility of a potential "sick-quitter" phenomenon (i.e., cessation of smoking might result from disease onset and changes in health conditions).[13,14] Therefore, for these reasons, we excluded all former smokers from the healthy group.

Finally, we defined never smoking as a healthy level in the present study.

1.2 Alcohol consumption

In the CLHLS, alcohol consumption is assessed through the following six self-report questions:

(1) Do you drink alcohol at present (response: yes/no)?
(2) Did you drink alcohol in the past (response: yes/no)?
(3) How old were you when you began drinking alcohol (response: age)?
(4) How old were you when stopped drinking alcohol if you don`t drink alcohol at present (response: age)?
(5) If you drink alcohol at the present time (or drank in the past), what kind of alcohol do (or did) you drink (response: very strong liquor [≥38% alcohol], not very strong liquor [<38% alcohol], wine, rice wine, beer, and others)?
(6) If you drink alcohol at the present time (or drank in the past), how much alcohol per day on average do (or did) you drink (response: Liang [Chinese Unit]; 1 Liang equals to 50 g)?

It was assumed that each participant drank one kind at a time, and the total amount of pure alcohol consumed daily could be calculated based on the alcohol content (v/v) of common Chinese alcoholic beverages:[15-17] very strong liquor (53%), not very strong liquor (35%), wine (12%), rice wine (15%), beer (4%), and others (24%, with an average alcohol content of the other five kinds). For example, if a participant drinks 1 Liang (50 g) of very strong liquor with a v/v of 53%, the amount of pure alcohol is calculated as follows:

- First, density of very strong liquor (v/v: 53%, 100 ml):

$$\begin{pmatrix} 1\frac{g}{ml} \times 47\ ml\ [density\ and\ volume\ for\ pure\ water] \\ + \\ 0.789\frac{g}{ml} \times 53\ ml\ [density\ and\ volume\ for\ pure\ alcohol] \end{pmatrix} \div 100\ ml = 0.888\frac{g}{ml}$$

- Second, volume for 50 g very strong liquor:

$$50\ g \div 0.888\frac{g}{ml} = 56.3\ ml$$

- Third, amount of pure alcohol for 50 g very strong liquor:

$$56.3\ ml \times 53\% \times 0.789\frac{g}{ml} = 23.5\ g$$

Therefore, the densities were 0.926 g/ml, 0.975 g/ml, 0.968 g/ml, 0.992 g/ml, and 0.949 g/ml for not very strong liquor (35%), wine (12%), rice wine (15%), beer (4%), and others (24%), respectively.

The dietary guidelines for Chinese residents (2022) recommend that adults should consume no more than 15 g of pure alcohol per day.[18] Additionally, in consideration of several studies indicating a higher mortality risk among former drinkers compared to never drinkers[14,19] and the potential sick-quitter phenomenon (i.e., cessation of alcohol consumption due to disease onset or changes in health conditions),[14] former drinkers who consumed more than 15 g of pure alcohol per day were excluded from the healthy group.

Finally, in the present study, a healthy level of alcohol consumption was defined as either never drinking or consuming ≤15 g of pure alcohol daily, both historically and currently—i.e., never or healthy drinking.

1.3 Physical activity

Based on the 2020 WHO guidelines on physical activity and sedentary behavior,[20] physical activity is defined as any bodily movement produced by skeletal muscles that requires energy expenditure, and its levels can be assessed in various domains, including leisure-time, occupation, education, household, and transportation, as well as exercise.

In the CLHLS, assessment of physical activity mainly includes two questions:

(1) Do you do exercises regularly at present (response: yes/no)?
(2) Do you now perform the following activities regularly? Across seven waves, these activities encompass seven forms of physical

activity and sedentary behavior (i.e., doing housework, engaging in personal outdoor activities [in the wave 1998: growing vegetables and other field work], doing garden work, raising domestic animals, reading newspapers/books, playing cards/mah-jong, and watching TV/listening to radio), with response options ranging from almost everyday to never.

Based on the two questions mentioned, we finally included five types of available physical activity: doing exercises, doing housework, engaging in personal outdoor activities, doing garden work, and raising domestic animals. The WHO guidelines[20] suggest older adults (aged 65 years and older) should do at least 150–300 minutes of moderate-intensity aerobic physical activity, or at least 75–150 minutes of vigorous-intensity aerobic physical activity, or an equivalent combination of moderate- and vigorous-intensity activity throughout the week, for substantial health benefits. However, information on the weekly duration of each specific type of physical activity is unavailable in the CLHLS. Given that older individuals are less likely to engage in prolonged periods of physical activity at one time, it is plausible that those who frequently engage in physical activity may meet the recommended duration of physical activity as per WHO guidelines.

Finally, for this study, we defined participants who exercised regularly (as mentioned in question 1) or engaged in physical activity almost everyday (as mentioned in question 2) as having a healthy level of physical activity.

1.4 Diet

In the CLHLS, self-reported dietary intake information is collected using a simplified food frequency questionnaire, which has been widely recognized and utilized in various studies.[21-23]

Based on the available food items across all seven waves of the CLHLS, a total of 12 food items were utilized to evaluate the healthful diet index for each participant. These 12 food items were categorized into three groups based on the previous studies:[23,24] healthful plant foods (staple food [coarse cereals], fresh fruit, fresh vegetables, beans, garlic, and tea), less healthful plant foods (staple food [rice, wheat flour], salt-preserved vegetables, and sugar), and animal foods (eggs, meat, and fish and aquatic products). Among the animal foods, numerous studies have demonstrated a negative association between higher consumption of eggs and meat and overall health outcomes,[25-29] indicating that these foods are less healthful; conversely, there is a positive association between the consumption of fish and other aquatic products and favorable health outcomes,[30-32] suggesting that the food is more healthful.

To calculate the healthful diet index, intake frequency scores were initially assigned to each food item: participants received positive intake frequency scores for each healthy food item but reverse scores for each less healthy food item (as shown in the table below). Then, the healthful diet index for each participant was calculated by summing the scores of the 12 food items, resulting in an index range between 13 and 49. A higher healthful diet index indicated a more healthful diet.

Finally, a healthy diet was defined as the health diet index in the top two-fifths of distribution.[10,33,34]

Table. Intake frequency score for each food item[a]

<table>
<tr><th></th><th>All seven waves</th><th>From wave 1998 to wave 2005</th><th>From wave 2008 to wave 2014</th></tr>
<tr><td>Healthful foods</td><td></td><td></td><td></td></tr>
<tr><td>Staple food [coarse cereals]</td><td>5</td><td></td><td></td></tr>
<tr><td>Fresh fruit</td><td rowspan="2">. almost every day =5,
. quite often/except winter =4,
. occasionally =2,
. rarely or never =1</td><td rowspan="2"></td><td rowspan="2"></td></tr>
<tr><td>Fresh vegetables</td></tr>
<tr><td>Beans</td><td rowspan="4"></td><td rowspan="4">. almost everyday =5,
. occasionally =2,
. rarely or never =1</td><td rowspan="4">. almost everyday =5,
. not everyday, but at least once per week =4,
. not every week, but at least once per month =3,
. not every month, but occasionally =2,
. rarely or never =1</td></tr>
<tr><td>Garlic</td></tr>
<tr><td>Tea</td></tr>
<tr><td>Fish and aquatic products</td></tr>
<tr><td>Less healthful foods</td><td></td><td></td><td></td></tr>
<tr><td>Staple food [rice, wheat flour]</td><td>1</td><td></td><td></td></tr>
<tr><td>Salt-preserved vegetables</td><td rowspan="4"></td><td rowspan="4">. almost everyday =1,
. occasionally =4,
. rarely or never =5</td><td rowspan="4">. almost everyday =1,
. not everyday, but at least once per week =2,
. not every week, but at least once per month =3,
. not every month, but occasionally =4,
. rarely or never =5</td></tr>
<tr><td>Sugar</td></tr>
<tr><td>Eggs</td></tr>
<tr><td>Meat</td></tr>
</table>

[a] For certain food items with varying response options across different waves, we assigned corresponding scores based on the response options.

2. Construction of healthy lifestyle score: weighted and simple methods

Given the interrelated nature of various lifestyle factors and their associations with overall survival, we constructed two types of healthy lifestyle scores: a weighted score and a simple score, and both scores encompassed cigarette smoking, alcohol consumption, physical activity, and diet. The two types of scores were applied to different analytical contexts.

Based on the previous studies,[35,36] a weighted healthy lifestyle score was constructed to account for varied magnitudes of the association between different lifestyle factors and overall survival. The weighted score was derived based on the β coefficients of each lifestyle factor within an accelerated failure model that included all four lifestyle factors while adjusting for sex, age, education, marital status, occupation prior to the age of 60 years, pension systems, residence, co-residence, and frailty status. For each lifestyle factor, the reference group (i.e., unhealthy level) was assigned a value of 0, and the β coefficient was assigned to the other category (i.e., healthy level). Subsequently, the assigned values were summed for each participant; then, the sum was divided by the total sum of the β coefficients, resulting in a weighted healthy lifestyle score for each participant, with the weighted score ranging from 0 to 1.

Furthermore, a simple method was used to construct another healthy lifestyle score.[10,13,37] For each lifestyle factor, a score of 1 was assigned for a healthy level and 0 for an unhealthy level. Finally, the total score was calculated as the sum of these scores, ranging between 0 and 4, with higher scores indicating higher adherence to healthy lifestyles. Although the underlying assumption was that the associations between different lifestyle factors and the outcome were identical, which might not be true, this simple method has been used widely and provides a more practical approach.

In general, the weighted healthy lifestyle score was used for mediation analysis on the association between frailty status and overall survival, while the simple healthy lifestyle score served as sensitivity analysis. Conversely, the simple healthy lifestyle score was used to examine the interactions and joint association of frailty status and lifestyles with overall survival, given its greater explanatory power and public significance; whereas the weighted healthy lifestyle score served as sensitivity analysis.

Supplementary Method 3: statistical analysis

1. Directed acyclic graphs

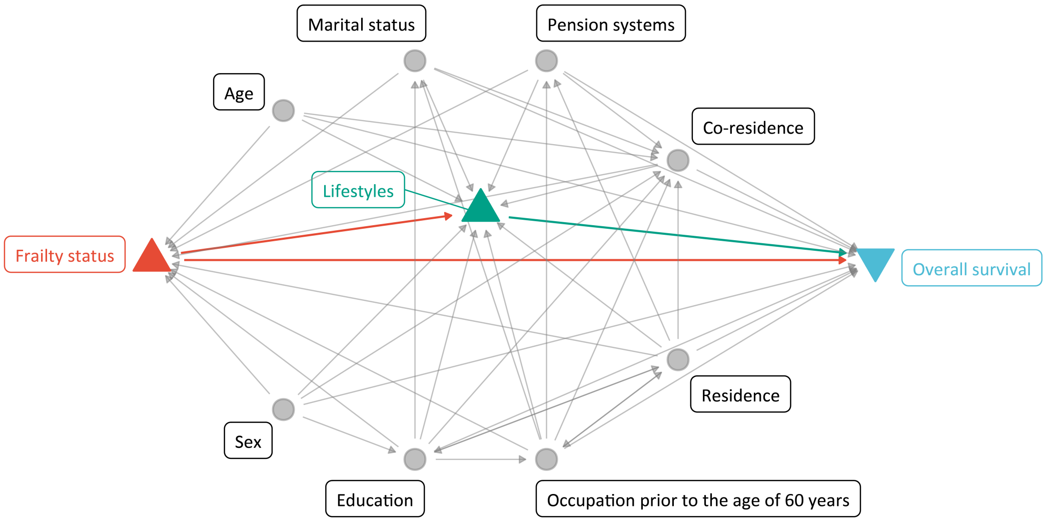

Note:
The directed acyclic graph provides a hypothesized theoretical framework to elucidate the association among exposures, mediators, covariates, and outcomes. However, one limitation of this method is that establishing causal associations among these elements can be challenging at times. Drawing on expert knowledge and previous studies regarding the associations among frailty status, lifestyles, covariates, and overall survival, the directed acyclic graph was developed for this study. Although some studies have indicated that unhealthy lifestyles contribute to the onset and progression of frailty,[38,39] it is conceptually and temporally more plausible to view frailty as a precursor to reduced physical activity (i.e., frailty → decreased activity → worse outcomes). Therefore, in the present study, the potential for bidirectional mediation was not taken into account during the mediation analysis.

Red node=frailty status (exposure), green node= lifestyles (mediator), blue node=overall survival (outcome), gray node=adjustment set used in the study.

2. Accelerated failure time models

Accelerated failure time models were used to evaluate the association of frailty status with overall survival, because Schoenfeld Residuals indicated a violation of proportional hazards assumption in the Cox proportional hazards models.[40,41] The model estimate the time ratio, which is interpreted as the expected time to events in one category relative to the reference group. Unlike interpreting proportional hazards model results where hazard ratios larger than 1 indicate higher risk, a time ratio greater than 1 is considered indicative of a longer time to events compared to the reference group.[40,41] Based on the minimum Akaike Information Criterion among different survival distributions (e.g., weibull, lognormal, logLogistic, and gaussian), we identified weibull distribution as most suitable for overall survival (as shown in the table below).

Table. Values of Akaike Information Criterion statistic of different distributions for survival time

	Values of Akaike Information Criterion statistic
Weibull	66205.6
Exponential	67699.8
Gaussian	76180.8
Logistic	75983.5
Lognormal	68216.7
LogLogistic	67209.2

3. Mediation analysis

Mediation analysis explores the mechanisms that underlie the observed association between an exposure variable and an outcome variable, while also examining their connection to a third intermediate variable known as the mediator.[42] Recently, significant advancements in mediation analysis have emerged through the application of the counterfactual framework, which has allowed for definitions of direct and indirect effects and for decomposition of a total effect into direct and indirect effects even in models with interactions and non-linearities.[42,43]

In this study, we performed a regression-based causal mediation analysis within the direct counterfactual framework to evaluate how healthy lifestyles mediate the association between frailty status and overall survival.[44,45] In brief, two regression models were fitted: one for the outcome (i.e., overall survival, using an accelerated failure model) and another for the mediator (i.e. weighted

healthy lifestyle score, using linear regression). In the two models, frailty status was treated as a categorical variable (robustness, pre-frailty, frailty), while the weighted healthy lifestyle score served as a linear term. Standard errors were estimated via bootstrapping with 1000 samples. All models were adjusted for potential confounders. The interaction between exposure and mediator was statistically insignificant; thus, results were calculated without considering the interaction.

Analyses were conducted in R (version 4.2.2) using the CMAverse package.[46]

4. Remaining life expectancy and years of life lost

We calculated the remaining life expectancy for participants of different levels of frailty status and simple healthy lifestyle scores after the specific age of 65 years and before the age of 100 years. To determine the remaining life expectancy, we employed a recently published method[47,48] that has been validated by other studies,[48-50] and a comprehensive guide on how to implement this method–utilizing a specific R package called "lillies"–has also been available.[51]

Moreover, we also calculated the years of life lost for participants of different levels of frailty status and simple healthy lifestyle scores in comparison to the reference population (i.e., those of robustness and four healthy lifestyle factors) of the same age at baseline. Finally, the relationship between age and years of life lost was smoothed using a linear regression model with a restricted cubic spline with three knots at the 10th, 50th, and 90th percentiles.

Importantly, the remaining life expectancy and years of life lost provided by "lillies" are intended to serve solely as a descriptive tool, rather than a basis for drawing causal conclusions.[51]

5. Multiple imputation

Evidence suggests that excluding participants without outcomes will tend to be a better strategy when fitting the outcome model (i.e., multiple imputation, then deletion).[52] Under the strategy, all participants are included in the imputation process; however, those for whom the outcome is imputed are excluded when fitting the outcome model. The strategy will tend to more efficient (i.e., reduced variability) than fitting the outcome model across all participants; in addition, the method is robust against bad imputation in the outcome.

In this analysis, multiple imputation was conducted on 42705 participants using chained equations to generate five datasets. Subsequently, 7320 participants with missing outcomes were excluded, and the remaining 35385 participants were used for the final analysis.

6. Cause-specific survival

The International Classification of Diseases, 10th Revision, was employed to evaluate the underlying cause of death among participants. Three waves (i.e., 2008, 2011, and 2014) did not provide details regarding the cause of death; therefore, we classified these deaths recorded during these waves as having an unknown cause.

Among the deaths, details of cause of death for 7815 participants were successfully obtained, including cardiovascular disease (CVD) mortality (n=1325, codes I00–I99) and non-CVD mortality (n=6490), and 5193 decedents were without a known cause of death. For assessing the association between frailty status and CVD-specific survival, survival status was categorized as "deceased" for participants who died from CVD, while it was defined as "censoring" for those who survived, died from non-CVD causes or had an unknown cause of death. A similar approach was used to assess the association between frailty status and non-CVD-specific survival.

In light of the fact that the three waves (i.e., 2008, 2011, and 2014) did not provide specific details regarding the causes of death, the analysis of cause-specific survival was considered exploratory in nature.

7. Study of osteoporotic fractures index

We conducted the sensitivity analysis by repeating the main analysis using the study of osteoporotic fractures (SOF) index as an indicator of frailty status.[53] In this study, we employed a modified SOF index based on data available from the CLHLS,[54,55] which comprised three components: (1) underweight (body mass index < 18.5 kg/m^2); (2) weak muscle strength (inability to stand up from a chair without the assistance of arms); and (3) low energy level (indicated by a positive response to the question "Over the last 6 months, have you been limited in activities because of a health problem?"). Subsequently, frailty status was categorised into three levels: robustness (0 components), pre-frailty (1 component), and frailty (2 or 3 components).[54,55]

Since the components of the SOF index were collected starting from wave 2011, only participants from waves 2011 to 2014 were included in the analysis (n=6629).

8. A 23-item frailty index

Conceptually and temporally, the impaired function captured by the frailty index should precede unhealthy lifestyles, particularly reduced physical activity (i.e., frailty → decreased physical activity → worse outcomes). However, it is essential to emphasize that the frailty index incorporates 12 specific items that are conceptually closely linked to physical activity. This inclusion may lead to potential overlap or interdependence between these items and physical activity. These items included ADLs, functional limitations, and the number of steps taken to complete a 360-degree turn without assistance. Although physical activities as lifestyle behaviors—such as doing exercises, doing housework, engaging in personal outdoor activities, doing garden work, and raising domestic animals—were not directly included in the 35-item frailty index developed for this study, bidirectional causality or residual overlap cannot be entirely ruled out; for instance, prolonged inactivity may also accelerate frailty. Consequently, we excluded these 12 items from the original 35-item frailty index and recalibrated it accordingly (resulting in a revised 23-item frailty index). Following this adjustment, we conducted the primary analysis again as a sensitivity analysis.

References

1. Rockwood K, Mitnitski A. Frailty in relation to the accumulation of deficits. *The journals of gerontology Series A, Biological sciences and medical sciences* 2007; 62(7): 722-7.
2. Theou O, Haviva C, Wallace L, Searle SD, Rockwood K. How to construct a frailty index from an existing dataset in 10 steps. *Age and ageing* 2023; 52(12).
3. Bennett S, Song X, Mitnitski A, Rockwood K. A limit to frailty in very old, community-dwelling people: a secondary analysis of the Chinese longitudinal health and longevity study. *Age and ageing* 2013; 42(3): 372-7.
4. Xu W, Liang Y, Lin Z. Association Between Neutrophil-Lymphocyte Ratio and Frailty: The Chinese Longitudinal Healthy Longevity Survey. *Frontiers in medicine* 2021; 8: 783077.
5. Zhang J, Wang Q, Hao W, Zhu D. Long-Term Food Variety and Dietary Patterns Are Associated with Frailty among Chinese Older Adults: A Cohort Study Based on CLHLS from 2014 to 2018. *Nutrients* 2022; 14(20).
6. Shrauner W, Lord EM, Nguyen XT, et al. Frailty and cardiovascular mortality in more than 3 million US Veterans. *European heart journal* 2022; 43(8): 818-26.
7. Orkaby AR, Nussbaum L, Ho YL, et al. The Burden of Frailty Among U.S. Veterans and Its Association With Mortality, 2002-2012. *The journals of gerontology Series A, Biological sciences and medical sciences* 2019; 74(8): 1257-64.
8. Ford ES, Zhao G, Tsai J, Li C. Low-risk lifestyle behaviors and all-cause mortality: findings from the National Health and Nutrition Examination Survey III Mortality Study. *Am J Public Health* 2011; 101(10): 1922-9.
9. World Health Organization. Tackling NCDs: 'Best buys' and other recommended interventions for the prevention and control of noncommunicable diseases. 2017. https://iris.who.int/bitstream/handle/10665/259232/WHO-NMH-NVI-17.9-eng.pdf?sequence=1&isAllowed=y (accessed Jul 6, 2024).
10. Zhang YB, Chen C, Pan XF, et al. Associations of healthy lifestyle and socioeconomic status with mortality and incident cardiovascular disease: two prospective cohort studies. *Bmj* 2021; 373: n604.
11. Yang JJ, Yu D, Shu XO, et al. Reduction in total and major cause-specific mortality from tobacco smoking cessation: a pooled analysis of 16 population-based cohort studies in Asia. *International journal of epidemiology* 2022; 50(6): 2070-81.
12. Wei Y, Lv Y, Zhou J, et al. Smoking cessation in late life is associated with increased risk of all-cause mortality amongst oldest old people: a community-based prospective cohort study. *Age and ageing* 2021; 50(4): 1298-305.
13. Sun Q, Yu D, Fan J, et al. Healthy lifestyle and life expectancy at age 30 years in the Chinese population: an observational study. *Lancet Public Health* 2022; 7(12): e994-e1004.
14. Xi B, Veeranki SP, Zhao M, Ma C, Yan Y, Mi J. Relationship of Alcohol Consumption to All-Cause, Cardiovascular, and Cancer-Related Mortality in U.S. Adults. *Journal of the American College of Cardiology* 2017; 70(8): 913-22.
15. Li J, Wu B, Tevik K, Krokstad S, Helvik AS. Factors associated with elevated consumption of alcohol in older adults-comparison between China and Norway: the CLHLS and the HUNT Study. *BMJ open* 2019; 9(8): e028646.
16. Wang Q, Zhang Y, Wu C. Alcohol consumption and associated factors among middle-aged and older adults: results from China Health and Retirement Longitudinal Study. *BMC public health* 2022; 22(1): 322.
17. Yang L, Zhou M, Sherliker P, et al. Alcohol drinking and overall and cause-specific mortality in China: nationally representative prospective study of 220,000 men with 15 years of follow-up. *International journal of epidemiology* 2012; 41(4): 1101-13.
18. Chinese Nutrition Society. Dietary Guidelines for Chinese Residents (2022). 2022. http://dg.cnsoc.org/upload/affix/20220427145839696.jpg (accessed Jul 6, 2024; in Chinese).
19. Stockwell T, Zhao J, Panwar S, Roemer A, Naimi T, Chikritzhs T. Do "Moderate" Drinkers Have Reduced Mortality Risk? A Systematic Review and Meta-Analysis of Alcohol Consumption and All-Cause Mortality. *J Stud Alcohol Drugs* 2016; 77(2): 185-98.
20. World Health Organization. WHO guidelines on physical activity and sedentary behaviour. 2020. https://www.who.int/publications/i/item/9789240015128 (accessed Jul 6, 2024).
21. Jin X, He W, Zhang Y, et al. Association of APOE epsilon4 genotype and lifestyle with cognitive function among Chinese adults aged 80 years and older: A cross-sectional study. *PLoS Med* 2021; 18(6): e1003597.
22. Zhang Y, Jin X, Lutz MW, et al. Interaction between APOE epsilon4 and dietary protein intake on cognitive decline: A longitudinal cohort study. *Clin Nutr* 2021; 40(5): 2716-25.
23. Chen H, Shen J, Xuan J, et al. Plant-based dietary patterns in relation to mortality among older adults in China. *Nat Aging* 2022; 2(3): 224-30.
24. Satija A, Bhupathiraju SN, Rimm EB, et al. Plant-Based Dietary Patterns and Incidence of Type 2 Diabetes in US Men and Women: Results from Three Prospective Cohort Studies. *PLoS Med* 2016; 13(6): e1002039.
25. Yang PF, Wang CR, Hao FB, et al. Egg consumption and risks of all-cause and cause-specific mortality: a dose-response meta-analysis of prospective cohort studies. *Nutr Rev* 2022; 80(7): 1739-54.
26. Zhao B, Gan L, Graubard BI, Mannisto S, Albanes D, Huang J. Associations of Dietary Cholesterol, Serum Cholesterol, and Egg Consumption With Overall and Cause-Specific Mortality: Systematic Review and Updated Meta-Analysis. *Circulation* 2022; 145(20): 1506-20.
27. Zhuang P, Wu F, Mao L, et al. Egg and cholesterol consumption and mortality from cardiovascular and different causes in the United States: A population-based cohort study. *PLoS Med* 2021; 18(2): e1003508.
28. Kennedy J, Alexander P, Taillie LS, Jaacks LM. Estimated effects of reductions in processed meat consumption and unprocessed red meat consumption on occurrences of type 2 diabetes, cardiovascular disease, colorectal cancer, and mortality in the USA: a microsimulation study. *Lancet Planet Health* 2024; 8(7): e441-e51.
29. Zhong VW, Van Horn L, Greenland P, et al. Associations of Processed Meat, Unprocessed Red Meat, Poultry, or Fish Intake With Incident Cardiovascular Disease and All-Cause Mortality. *JAMA Intern Med* 2020; 180(4): 503-12.

30. Bakre AT, Chen A, Tao X, et al. Impact of fish consumption on all-cause mortality in older people with and without dementia: a community-based cohort study. *Eur J Nutr* 2022; 61(7): 3785-94.
31. Jiang L, Wang J, Xiong K, Xu L, Zhang B, Ma A. Intake of Fish and Marine n-3 Polyunsaturated Fatty Acids and Risk of Cardiovascular Disease Mortality: A Meta-Analysis of Prospective Cohort Studies. *Nutrients* 2021; 13(7).
32. Sun H. Fish and Shellfish Consumption, Cognitive Health and Mortality from Alzheimer's Disease among US Adults Aged 60 and Older. *J Prev Alzheimers Dis* 2024; 11(3): 632-8.
33. Li Y, Pan A, Wang DD, et al. Impact of Healthy Lifestyle Factors on Life Expectancies in the US Population. *Circulation* 2018; 138(4): 345-55.
34. Li Y, Schoufour J, Wang DD, et al. Healthy lifestyle and life expectancy free of cancer, cardiovascular disease, and type 2 diabetes: prospective cohort study. *Bmj* 2020; 368: l6669.
35. Wang J, Chen C, Zhou J, et al. Healthy lifestyle in late-life, longevity genes, and life expectancy among older adults: a 20-year, population-based, prospective cohort study. *Lancet Healthy Longev* 2023; 4(10): e535-e43.
36. Zhong J, Zhang Y, Zhu K, et al. Associations of social determinants of health with life expectancy and future health risks among individuals with type 2 diabetes: two nationwide cohort studies in the UK and USA. *Lancet Healthy Longev* 2024; 5(8): e542-e51.
37. Zhang Y, Pan XF, Chen J, et al. Combined lifestyle factors and risk of incident type 2 diabetes and prognosis among individuals with type 2 diabetes: a systematic review and meta-analysis of prospective cohort studies. *Diabetologia* 2020; 63(1): 21-33.
38. Hoogendijk EO, Afilalo J, Ensrud KE, Kowal P, Onder G, Fried LP. Frailty: implications for clinical practice and public health. *Lancet* 2019; 394(10206): 1365-75.
39. Haapanen MJ, Mikkola TM, Jylhava J, et al. Lifestyle-related factors in late midlife as predictors of frailty from late midlife into old age: a longitudinal birth cohort study. *Age and ageing* 2024; 53(4).
40. Gregson J, Sharples L, Stone GW, Burman CF, Ohrn F, Pocock S. Nonproportional Hazards for Time-to-Event Outcomes in Clinical Trials: JACC Review Topic of the Week. *Journal of the American College of Cardiology* 2019; 74(16): 2102-12.
41. Ananthakrishnan R, Green S, Previtali A, Liu R, Li D, LaValley M. Critical review of oncology clinical trial design under non-proportional hazards. *Crit Rev Oncol Hematol* 2021; 162: 103350.
42. Valeri L, Vanderweele TJ. Mediation analysis allowing for exposure-mediator interactions and causal interpretation: theoretical assumptions and implementation with SAS and SPSS macros. *Psychol Methods* 2013; 18(2): 137-50.
43. Valente MJ, Rijnhart JJM, Smyth HL, Muniz FB, MacKinnon DP. Causal Mediation Programs in R, Mplus, SAS, SPSS, and Stata. *Struct Equ Modeling* 2020; 27(6): 975-84.
44. VanderWeele TJ. Causal mediation analysis with survival data. *Epidemiology* 2011; 22(4): 582-5.
45. Li Y, Yoshida K, Kaufman JS, Mathur MB. A brief primer on conducting regression-based causal mediation analysis. *Psychol Trauma* 2023; 15(6): 930-8.
46. Shi B, Choirat C, Coull BA, VanderWeele TJ, Valeri L. CMAverse: A Suite of Functions for Reproducible Causal Mediation Analyses. *Epidemiology* 2021; 32(5): e20-e2.
47. Andersen PK. Life years lost among patients with a given disease. *Statistics in medicine* 2017; 36(22): 3573-82.
48. Erlangsen A, Andersen PK, Toender A, Laursen TM, Nordentoft M, Canudas-Romo V. Cause-specific life-years lost in people with mental disorders: a nationwide, register-based cohort study. *Lancet Psychiatry* 2017; 4(12): 937-45.
49. Chang WH, Lai AG. Cumulative burden of psychiatric disorders and self-harm across 26 adult cancers. *Nat Med* 2022; 28(4): 860-70.
50. Plana-Ripoll O, Dreier JW, Momen NC, et al. Analysis of mortality metrics associated with a comprehensive range of disorders in Denmark, 2000 to 2018: A population-based cohort study. *PLoS Med* 2022; 19(6): e1004023.
51. Plana-Ripoll O, Canudas-Romo V, Weye N, Laursen TM, McGrath JJ, Andersen PK. lillies: An R package for the estimation of excess Life Years Lost among patients with a given disease or condition. *PloS one* 2020; 15(3): e0228073.
52. von Hippel PT. Regression with Missing Ys: An Improved Strategy for Analyzing Multiply Imputed Data. *Sociological Methodology* 2007; 37(1): 83-117.
53. Ensrud KE, Ewing SK, Taylor BC, et al. Comparison of 2 frailty indexes for prediction of falls, disability, fractures, and death in older women. *Arch Intern Med* 2008; 168(4): 382-9.
54. Liu L, Chen C, Lo K, et al. Serum 25-hydroxyvitamin D, frailty, and mortality among the Chinese oldest old: Results from the CLHLS study. *Nutrition, Metabolism and Cardiovascular Diseases* 2021; 31(9): 2707-15.
55. Lv YB, Gao X, Yin ZX, et al. Revisiting the association of blood pressure with mortality in oldest old people in China: community based, longitudinal prospective study. *Bmj* 2018; 361: k2158.

Supplementary Table 1. Definitions of baseline covariates in the present study

	Questions<br>in the CLHLS questionnaire	Options for the questions	Scales of reclassification in the present study
Sex		. male<br>. female	. Male: male<br>. Female: female
Age			. Continuous (years)
Education	How many years did you attend school?	. years of school<br>. don't know<br>. missing	. No school: years of school =0<br>. 1 year or more: years of school ≥1<br>. missing: don't know, missing
Marital status	Current marital status?	. currently married and living with spouse<br>. separated<br>. divorced<br>. widowed<br>. never married<br>. don't know<br>. missing	. Not in marriage: separated, divorced, widowed, never married<br>. In marriage: currently married and living with spouse<br>. missing: don't know, missing
Occupations before the age of 60 years	What was your primary occupation before the age of 60 years?	Waves 1998 and 2000<br>. professional and technical personnel<br>. governmental, institutional or managerial personnel<br>. agriculture, forest, animal husbandry<br>. fishery worker<br>. industrial worker<br>. commercial or service worker<br>. military personnel<br>. housework<br>. others<br>. missing<br><br>Waves 2002, 2005, 2008, 2011, and 2014<br>. professional and technical personnel<br>. governmental, institutional or managerial personnel<br>. staff/service workers/industrial workers<br>. self-employed<br>. personnel in agriculture fishery forestry animal husbandry<br>. housework<br>. military personnel<br>. unemployed<br>. others<br>. missing	Since all participants in our study were retired (age ≥65 years), we used the primary occupation before the age of 60 years as the occupational levels. Based on the previous studies (①Am J Respir Crit Care Med 2013;187(3):303-310; ②J Epidemiol Glob Health 2023;13(2):322-332), occupations were categorized into three levels:<br>. Low occupational grade: agriculture, forestry, animal husbandry, fishery, housework, unemployed or others<br>. Medium occupational grade: staff, service/industrial workers, self-employed or military personnel<br>. High occupational grade: governmental/institutional/managerial personnel, and professional/technical personnel<br>. missing: missing
Pension systems	What is your primary means of financial support?	Waves 1998, 2000, and 2002<br>. retirement wages<br>. spouse	. Without pensions: spouse, child(ren), grandchild(ren), relative(s), local government or community, work, other, no other means

	Questions in the CLHLS questionnaire	Options for the questions	Scales of reclassification in the present study
		. child(ren)<br>. grandchild(ren)<br>. relative(s)<br>. local government or community<br>. work<br>. other<br>. missing<br><br>Waves 2005, 2008, 2011, and 2014<br>. retirement wages<br>. spouse<br>. child(ren)<br>. grandchild(ren)<br>. relative(s)<br>. local government or community<br>. work<br>. other<br>. no other means<br>. missing	. With pensions: retirement wages<br>. missing: missing
Residence	Current residence area of interviewees?	Wave 1998<br>. urban (city and town)<br>. rural<br><br>Waves 2000, 2002, 2005, 2008, 2011, and 2014<br>. city<br>. town<br>. rural	. Rural: rural<br>. Urban: city, town
Co-residence	Co-residence?	. with family member(s)<br>. alone<br>. in a nursing home<br>. missing	. With family members: with household member(s)<br>. Alone: alone<br>. In an institution: in a nursing home<br>. missing: missing

More detailed information about these covariates can be found on: https://agingcenter.duke.edu/CLHLS.
Abbreviations: CLHLS=Chinese Longitudinal Healthy Longevity Surveys.

Supplementary Table 2. Distributions of baseline covariates with missing data

	Number of missing data	Percentage of missing data (%)
Sex	0	0.00
Age	0	0.00
Education	108	0.51
Marital status	8	0.04
Occupation before the age of 60 years	61	0.29
Pension systems	3	0.01
Residence	0	0.00
Co-residence	5	0.02

Missing data were assumed to be completely random, and participants with missing information were excluded from the main analyses. Nevertheless, we also conducted multiple imputation as a sensitivity analysis.

Supplementary Table 3. Baseline characteristics of participants included or excluded from analyses

	All	Included participants	Excluded participants[a]	p value
No. of participants	43247	17476	25771	
Sex: male	17727 (41.0%)	10753 (61.5%)	6974 (27.1%)	<0.001
Age (years)	90.0 (80.0–99.0)	87.0 (80.0–95.0)	92.0 (81.0–100.0)	<0.001
Education				<0.001
No school	27901 (65.0%)	9560 (54.7%)	18341 (72.0%)	
1 year or more	15042 (35.0%)	7916 (45.3%)	7126 (28.0%)	
Marital status				<0.001
Not in marriage	31846 (73.7%)	11690 (66.9%)	20156 (78.3%)	
In marriage	11369 (26.3%)	5786 (33.1%)	5583 (21.7%)	
Occupation before the age of 60 years				<0.001
Low occupational grade	33121 (77.0%)	12492 (71.5%)	20629 (80.7%)	
Medium occupational grade	6926 (16.1%)	3328 (19.0%)	3598 (14.1%)	
High occupational grade	2992 (7.0%)	1656 (9.5%)	1336 (5.2%)	
Pension systems				<0.001
Without pensions	35946 (83.1%)	13777 (78.8%)	22169 (86.1%)	
With pensions	7287 (16.9%)	3699 (21.2%)	3588 (13.9%)	
Residence				<0.001
Rural	25076 (58.0%)	9926 (56.8%)	15150 (58.8%)	
Urban	18171 (42.0%)	7550 (43.2%)	10621 (41.2%)	
Co-residence				<0.001
With family members	36052 (83.4%)	14743 (84.4%)	21309 (82.8%)	
Alone	5540 (12.8%)	2070 (11.8%)	3470 (13.5%)	
In an institution	1625 (3.8%)	663 (3.8%)	962 (3.7%)	
Comorbidities				
Hypertension	6736 (16.4%)	2634 (15.1%)	4102 (17.3%)	<0.001
Diabetes	821 (2.0%)	294 (1.7%)	527 (2.2%)	<0.001
Heart disease	3428 (8.3%)	1353 (7.7%)	2075 (8.8%)	<0.001
Stroke or cerebrovascular disease	1984 (4.8%)	713 (4.1%)	1271 (5.4%)	<0.001
Bronchitis, emphysema, pneumonia, asthma	4855 (11.7%)	2128 (12.2%)	2727 (11.4%)	0.018
Tuberculosis	290 (0.7%)	133 (0.8%)	157 (0.7%)	0.256
Cancer	184 (0.5%)	72 (0.4%)	112 (0.5%)	0.359
Gastric or duodenal ulcer	1831 (4.6%)	696 (4.0%)	1135 (5.1%)	<0.001
Parkinson's disease	241 (0.6%)	103 (0.6%)	138 (0.6%)	0.944
Bedsore	312 (0.8%)	96 (0.5%)	216 (1.0%)	<0.001
Cataract	5564 (13.5%)	2027 (11.6%)	3537 (14.9%)	<0.001
Glaucoma	981 (2.5%)	329 (1.9%)	652 (2.9%)	<0.001
Prostate tumor	1172 (4.3%)	658 (3.8%)	514 (5.2%)	<0.001
Never smoking	29548 (68.4%)	9956 (57.0%)	19592 (76.2%)	<0.001
Never or healthy drinking	33554 (78.4%)	12255 (70.1%)	21299 (84.2%)	<0.001
Healthy physical activity	25306 (58.6%)	11392 (65.2%)	13914 (54.1%)	<0.001
Healthy diet	17511 (41.1%)	7643 (43.7%)	9868 (39.3%)	<0.001
Simple healthy lifestyle score	3.0 (2.0–3.0)	2.0 (2.0–3.0)	3.0 (2.0–3.0)	<0.001
Weighted healthy lifestyle score	0.58 (0.36–0.86)	0.58 (0.36–0.86)	0.58 (0.36–0.86)	0.109
Frailty index	0.11 (0.08–0.16)	0.11 (0.08–0.16)	0.11 (0.08–0.17)	<0.001

Values are median (IQR) or n (%).

[a] Each variable was shown by the actual sample size.

Abbreviations: IQR = interquartile range.

Supplementary Table 4. Associations between frailty status and overall survival

	Robustness	pre-Frailty	Frailty
No. of participants	7766	7116	2594
No. of deaths	5164	5528	2316
Crude TR (95% CI), p	1.00 (ref)	0.63 (0.61–0.65)	0.35 (0.34–0.37)
Adjusted TR (95% CI), p			
model 1[a]	1.00 (ref)	0.84 (0.81–0.86)	0.59 (0.57–0.62)
model 2[b]	1.00 (ref)	0.84 (0.82–0.86)	0.59 (0.57–0.62)
model 3[c]	1.00 (ref)	0.86 (0.83–0.88)	0.64 (0.61–0.66)

[a] Model 1 with adjustment for sex and age.

[b] Model 2 with adjustment for covariates in model 1 and additional factors such as education, marital status, occupation before the age of 60 years, pension systems, residence, and co-residence. Overall survival showed a significantly shorter trend with increased frailty severity (p-value for trend <0.001), and the p-value for this trend was derived from Wald tests of a linear association of frailty severity as a numeral (robustness =1, pre-frailty =2, and frailty =3) and overall survival.

[c] Model 3 with adjustment for covariates in model 2 plus weighted healthy lifestyle score.

Abbreviations: CI=confidence interval, TR=time ratio.

Supplementary Table 5. Associations between healthy lifestyle score, as well as each healthy lifestyle factor, and overall survival

	Weighted healthy lifestyle score (as a continuous variable)	Simple healthy lifestyle score [d] (as a continuous variable)	Never smoking	Never or healthy drinking	Healthy physical activity	Healthy diet
Changes	per 0.1 increase	per 1 increase	yes vs. no	yes vs. no	yes vs. no	yes vs. no
No. of participants	17476	17476	9956 vs. 7520	12255 vs. 5221	11392 vs. 6084	7643 vs. 9833
No. of deaths	13008	13008	7558 vs. 5450	9160 vs. 3848	7759 vs. 5249	5403 vs. 7605
Crude TR (95% CI), p	1.105 (1.098–1.111)	1.165 (1.147–1.184)				
Adjusted TR (95% CI), p						
model 1[a]	1.051 (1.046–1.056)	1.117 (1.102–1.132)				
model 2[b]	1.048 (1.043–1.054)	1.109 (1.094–1.124)				
model 3[c]	1.034 (1.029–1.039)	1.082 (1.068–1.097)	1.10 (1.06–1.13)	1.03 (1.00–1.06)	1.18 (1.15–1.22)	1.05 (1.02–1.07)

[a] Model 1 with adjustment for sex and age.

[b] Model 2 with adjustment for covariates in model 1 and additional factors such as education, marital status, occupation before the age of 60 years, pension systems, residence, and co-residence.

[c] Model 3 with adjustment for covariates in model 2 plus frailty status, and for each healthy lifestyle factor, further adjustments were made for the other remaining healthy lifestyle factors.

[d] When the simple healthy lifestyle score was considered as a categorical variable, the adjusted TRs were 1.12 (1.04–1.20) for a score of 1, 1.22 (1.14–1.30) for a score of 2, 1.32 (1.23–1.41) for a score of 3, and 1.39 (1.29–1.50) for a score of 4, with a score of 0 serving as the reference.

Abbreviations: CI=confidence interval, TR=time ratio.

Supplementary Table 6. Linear regression for the association between frailty index and healthy lifestyle score

	Frailty index (as a categorical variable)			Frailty index (as a continuous variable), per 0.1 increase
	Robustness	pre-Frailty	Frailty	
No. of participants	7766	7116	2594	17476
Weighted healthy lifestyle score				
Crude coefficient (95% CI), p	1.00 (ref)	-0.08 (-0.09 to -0.07)	-0.25 (-0.26 to -0.23)	
Adjusted coefficient (95% CI), p				
model 1[a]	1.00 (ref)	-0.07 (-0.08 to -0.06)	-0.22 (-0.23 to -0.21)	
model 2[b]	1.00 (ref)	-0.07 (-0.08 to -0.06)	-0.22 (-0.23 to -0.21)	-0.096 (-0.101 to -0.091)
Simple healthy lifestyle score				
Crude coefficient (95% CI), p	1.00 (ref)	-0.14 (-0.18 to -0.11)	-0.44 (-0.48 to -0.40)	
Adjusted coefficient (95% CI), p				
model 1[a]	1.00 (ref)	-0.17 (-0.20 to -0.13)	-0.49 (-0.53 to -0.44)	
model 2[b]	1.00 (ref)	-0.16 (-0.20 to -0.13)	-0.49 (-0.54 to -0.45)	-0.215 (-0.233 to -0.196)

[a] Model 1 with adjustment for sex and age.

[b] Model 2 with adjustment for covariates in model 1 and additional factors such as education, marital status, occupation before the age of 60 years, pension systems, residence, and co-residence.

Abbreviations: CI=confidence interval.

Supplementary Table 7. Mediation analysis of lifestyles on the association between frailty status and overall survival: subgroup analysis

Association[a]	Robustness	pre-Frailty	Frailty
Sex: male (n=10753)[b]			
Total effect; TR (95% CI)	1.00 (ref)	0.82 (0.79–0.85)	0.57 (0.54–0.61)
Natural direct effect; TR (95% CI)		0.84 (0.81–0.87)	0.62 (0.58–0.66)
Natural indirect effect; TR (95% CI)		0.978 (0.972–0.983)	0.928 (0.914–0.942)
Mediation proportion; % (95% CI)		10.4 (7.6–14.4)	10.5 (7.8–13.3)
Sex: female (n=6723)[b]			
Total effect; TR (95% CI)	1.00 (ref)	0.87 (0.83–0.91)	0.61 (0.57–0.65)
Natural direct effect; TR (95% CI)		0.89 (0.85–0.93)	0.66 (0.62–0.70)
Natural indirect effect; TR (95% CI)		0.975 (0.967–0.983)	0.927 (0.911–0.944)
Mediation proportion; % (95% CI)		17.1 (10.8–27.0)	12.2 (8.9–16.0)
Age: <80 years (n=3910)[c]			
Total effect; TR (95% CI)	1.00 (ref)	0.79 (0.73–0.85)	0.51 (0.44–0.59)
Natural direct effect; TR (95% CI)		0.80 (0.74–0.86)	0.54 (0.47–0.63)
Natural indirect effect; TR (95% CI)		0.989 (0.981–0.995)	0.935 (0.902–0.964)
Mediation proportion; % (95% CI)		4.1 (1.6–8.4)	7.2 (4.0–11.9)
Age: ≥80 years (n=13566)[c]			
Total effect; TR (95% CI)	1.00 (ref)	0.77 (0.74–0.80)	0.48 (0.46–0.50)
Natural direct effect; TR (95% CI)		0.80 (0.77–0.83)	0.53 (0.51–0.56)
Natural indirect effect; TR (95% CI)		0.962 (0.956–0.969)	0.898 (0.885–0.911)
Mediation proportion; % (95% CI)		13.0 (10.4–16.1)	10.4 (8.6–12.1)

[a] Natural direct effect and natural indirect effect estimated the effect of frailty status on overall survival that did not or did act through the mediator (i.e., lifestyles, measured by weighted healthy lifestyle score), respectively. The mediation proportion estimated the percentage of the effect of frailty status, on the log(TR) scale, that acted through the mediator (i.e., lifestyles, measured by weighted healthy lifestyle score).

[b] The model was adjusted for age, education, marital status, occupation prior to the age of 60 years, pension systems, residence, and co-residence.

[c] The model was adjusted for sex, education, marital status, occupation prior to the age of 60 years, pension systems, residence, and co-residence.

Abbreviations: CI=confidence interval, TR=time ratio.

Supplementary Table 8. Mediation analysis of lifestyles on the association between frailty status and overall survival: reverse causation, censoring losses at different time points, and without comorbidities

Association[a]	Robustness	pre-Frailty	Frailty
Excluding deaths within the first year (n=15426)			
Total effect; TR (95% CI)	1.00 (ref)	0.88 (0.86–0.90)	0.68 (0.65–0.71)
Natural direct effect; TR (95% CI)		0.89 (0.87–0.92)	0.71 (0.69–0.75)
Natural indirect effect; TR (95% CI)		0.985 (0.982–0.989)	0.950 (0.940–0.960)
Mediation proportion; % (95% CI)		10.6 (7.8–14.8)	11.1 (8.6–13.8)
Losses censored at the median follow-up time (n=20945)			
Total effect; TR (95% CI)	1.00 (ref)	0.86 (0.84–0.88)	0.68 (0.65–0.71)
Natural direct effect; TR (95% CI)		0.88 (0.85–0.90)	0.73 (0.70–0.76)
Natural indirect effect; TR (95% CI)		0.980 (0.976–0.983)	0.935 (0.925–0.945)
Mediation proportion; % (95% CI)		12.6 (9.8–16.2)	14.7 (12.2–17.8)
Losses censored at the mean follow-up time (n=20945)			
Total effect; TR (95% CI)	1.00 (ref)	0.87 (0.84–0.89)	0.71 (0.68–0.74)
Natural direct effect; TR (95% CI)		0.88 (0.86–0.91)	0.76 (0.73–0.80)
Natural indirect effect; TR (95% CI)		0.980 (0.976–0.984)	0.935 (0.924–0.945)
Mediation proportion; % (95% CI)		13.3 (10.2–17.4)	17.2 (13.9–21.1)
Without comorbidities[b] (n=9903)			
Total effect; TR (95% CI)	1.00 (ref)	0.81 (0.78–0.84)	0.56 (0.52–0.61)
Natural direct effect; TR (95% CI)		0.83 (0.80–0.87)	0.61 (0.56–0.66)
Natural indirect effect; TR (95% CI)		0.972 (0.963–0.979)	0.926 (0.908–0.943)
Mediation proportion; % (95% CI)		12.5 (8.7–17.8)	10.3 (7.3–13.7)

[a] Natural direct effect and natural indirect effect estimated the effect of frailty status on overall survival that did not or did act through the mediator (i.e., lifestyles, measured by weighted healthy lifestyle score), respectively. The mediation proportion estimated the percentage of the effect of frailty status, on the log(TR) scale, that acted through the mediator (i.e., lifestyles, measured by weighted healthy lifestyle score). The model was adjusted for sex, age, education, marital status, occupation prior to the age of 60 years, pension systems, residence, and co-residence.

[b] If participants were free of all comorbidities listed in Table 1, they were classified as "without comorbidities".

Abbreviations: CI=confidence interval, TR=time ratio.

Supplementary Table 9. Mediation analysis of lifestyles on the association between frailty status and survival: multiple imputation and cause-specific survival

Association[a]	Robustness	pre-Frailty	Frailty
Multiple imputation[b] (n=35385)			
Total effect; TR (95% CI)	1.00 (ref)	0.84 (0.82–0.86)	0.62 (0.60–0.64)
Natural direct effect; TR (95% CI)		0.86 (0.84–0.88)	0.67 (0.65–0.69)
Natural indirect effect; TR (95% CI)		0.973 (0.969–0.976)	0.920 (0.912–0.927)
Mediation proportion; % (95% CI)		14.4 (12.1–17.2)	14.3 (12.5–16.1)
CVD-specific survival[c] (n=17476)			
Total effect; TR (95% CI)	1.00 (ref)	0.69 (0.61–0.80)	0.34 (0.29–0.41)
Natural direct effect; TR (95% CI)		0.72 (0.64–0.83)	0.39 (0.33–0.47)
Natural indirect effect; TR (95% CI)		0.961 (0.948–0.975)	0.877 (0.839–0.917)
Mediation proportion; % (95% CI)		9.2 (5.3–17.2)	7.4 (4.6–11.3)
non-CVD-specific survival[c] (n=17476)			
Total effect; TR (95% CI)	1.00 (ref)	0.79 (0.75–0.84)	0.52 (0.49–0.56)
Natural direct effect; TR (95% CI)		0.83 (0.78–0.87)	0.60 (0.56–0.64)
Natural indirect effect; TR (95% CI)		0.960 (0.952–0.968)	0.876 (0.859–0.894)
Mediation proportion; % (95% CI)		15.9 (11.7–21.8)	15.5 (12.5–19.1)

[a] Natural direct effect and natural indirect effect estimated the effect of frailty status on survival that did not or did act through the mediator (i.e., lifestyles, measured by weighted healthy lifestyle score), respectively. The mediation proportion estimated the percentage of the effect of frailty status, on the log(TR) scale, that acted through the mediator (i.e., lifestyles, measured by weighted healthy lifestyle score). The model was adjusted for sex, age, education, marital status, occupation prior to the age of 60 years, pension systems, residence, and co-residence.

[b] We used imputed dataset 1 to assess the association, and the results obtained from imputations 2 to 5 were similar (data not shown). For detailed information regarding multiple imputation, please refer to Supplementary Method 3.

[c] For detailed information regarding cause-specific survival, please refer to Supplementary Method 3.

Abbreviations: CI=confidence interval, CVD=cardiovascular disease, TR=time ratio.

Supplementary Table 10. Mediation analysis of lifestyles on the association between frailty status and overall survival: several additional methodologies for mediation analysis (n=17476)

Association[a]	Robustness	pre-Frailty	Frailty
Regression-based approach[b]			
Total effect; TR (95% CI)	1.00 (ref)	0.84 (0.81–0.86)	0.59 (0.57–0.61)
Natural direct effect; TR (95% CI)		0.86 (0.83–0.88)	0.64 (0.61–0.66)
Natural indirect effect; TR (95% CI)		0.978 (0.973–0.982)	0.929 (0.918–0.939)
Mediation proportion; % (95% CI)		11.9 (8.8–15.1)	11.1 (9.0–13.1)
G-formula approach[c]			
Total effect; TR (95% CI)	1.00 (ref)	0.84 (0.82–0.86)	0.59 (0.57–0.62)
Natural direct effect; TR (95% CI)		0.86 (0.83–0.88)	0.64 (0.61–0.66)
Natural indirect effect; TR (95% CI)		0.978 (0.973–0.982)	0.929 (0.918–0.938)
Mediation proportion; % (95% CI)		11.9 (9.2–15.3)	11.1 (9.2–13.3)

[a] Natural direct effect and natural indirect effect estimated the effect of frailty status on overall survival that did not or did act through the mediator (i.e., lifestyles, measured by weighted healthy lifestyle score), respectively. The mediation proportion estimated the percentage of the effect of frailty status, on the log(TR) scale, that acted through the mediator (i.e., lifestyles, measured by weighted healthy lifestyle score). The model was adjusted for sex, age, education, marital status, occupation prior to the age of 60 years, pension systems, residence, and co-residence.

[b] Causal effect was estimated through the closed-form parameter function estimation, and standard errors of causal effect were estimated through the delta method.

[c] Causal effect was estimated through the direct counterfactual imputation estimation, and standard errors of causal effect were estimated through the bootstrapping.

Abbreviations: CI=confidence interval, TR=time ratio.

Supplementary Table 11. Mediation analysis of lifestyles on the association between frailty status and overall survival: alternative methodologies used for the mediator and the frailty status (n=17476)

Association[a]	Robustness	pre-Frailty	Frailty	
Healthy lifestyle factors as multiple mediators[b]				
Total effect; TR (95% CI)	1.00 (ref)	0.84 (0.82–0.87)	0.60 (0.57–0.62)	
Natural direct effect; TR (95% CI)		0.86 (0.83–0.88)	0.64 (0.61–0.66)	
Natural indirect effect; TR (95% CI)		0.983 (0.977–0.985)	0.938 (0.925–0.947)	
Mediation proportion; % (95% CI)		9.3 (7.4–13.4)	9.7 (8.0–12.3)	
Simple healthy lifestyle score as a mediator				
Total effect; TR (95% CI)	1.00 (ref)	0.84 (0.81–0.86)	0.59 (0.57–0.62)	
Natural direct effect; TR (95% CI)		0.85 (0.82–0.87)	0.61 (0.59–0.64)	
Natural indirect effect; TR (95% CI)		0.987 (0.984–0.990)	0.962 (0.955–0.969)	
Mediation proportion; % (95% CI)		6.7 (4.7–8.8)	5.7 (4.5–6.9)	
Frailty index as a continuous variable[c]				
Total effect; TR (95% CI)				0.79 (0.78–0.80)
Natural direct effect; TR (95% CI)				0.81 (0.80–0.83)
Natural indirect effect; TR (95% CI)				0.970 (0.965–0.975)
Mediation proportion; % (95% CI)				11.5 (9.5–13.6)

[a] Natural direct effect and natural indirect effect estimated the effect of frailty status on overall survival that did not or did act through the mediator, respectively. The mediation proportion estimated the percentage of the effect of frailty status, on the log(TR) scale, that acted through the mediator. The model was adjusted for sex, age, education, marital status, occupation prior to the age of 60 years, pension systems, residence, and co-residence.

[b] Four healthy lifestyle factors (i.e, never smoking, never or healthy drinking, healthy physical activity, and healthy diet) were included as multiple mediators.

[c] Frailty index was considered as a continuous variable, and TR was given per 0.1 increase of frailty index.

Abbreviations: CI=confidence interval, TR=time ratio.

Supplementary Table 12. Mediation analysis of lifestyles on the association between frailty status and overall survival: using the study of osteoporotic fractures index or the 23-item frailty index to assess frailty status

Association[a]	Robustness	pre-Frailty	Frailty
Study of osteoporotic fractures index (n=6629)			
Total effect; TR (95% CI)	1.00 (ref)	0.72 (0.68–0.77)	0.54 (0.50–0.58)
Natural direct effect; TR (95% CI)		0.74 (0.69–0.79)	0.59 (0.55–0.64)
Natural indirect effect; TR (95% CI)		0.980 (0.972–0.987)	0.914 (0.893–0.934)
Mediation proportion; % (95% CI)		5.4 (3.3–8.5)	11.0 (8.1–14.7)
23-item frailty index (n=17476)			
Total effect; TR (95% CI)	1.00 (ref)	0.86 (0.83–0.89)	0.71 (0.68–0.74)
Natural direct effect; TR (95% CI)		0.88 (0.85–0.91)	0.75 (0.71–0.78)
Natural indirect effect; TR (95% CI)		0.977 (0.972–0.981)	0.944 (0.936–0.952)
Mediation proportion; % (95% CI)		14.6 (11.2–19.4)	14.2 (11.6–17.2)

[a] Natural direct effect and natural indirect effect estimated the effect of frailty status on overall survival that did not or did act through the mediator (i.e., lifestyles, measured by weighted healthy lifestyle score), respectively. The mediation proportion estimated the percentage of the effect of frailty status, on the log(TR) scale, that acted through the mediator (i.e., lifestyles, measured by weighted healthy lifestyle score). The model was adjusted for sex, age, education, marital status, occupation prior to the age of 60 years, pension systems, residence, and co-residence.

Abbreviations: CI=confidence interval, TR=time ratio.

Supplementary Table 13. Mediation analysis of lifestyles on the association between frailty status and overall survival: each lifestyle factor (n=17476)

Association[a]	Robustness	pre-Frailty	Frailty
Never smoking			
Total effect; TR (95% CI)	1.00 (ref)	0.86 (0.83–0.88)	0.64 (0.61–0.66)
Natural direct effect; TR (95% CI)		0.86 (0.83–0.88)	0.64 (0.61–0.67)
Natural indirect effect; TR (95% CI)		1.000 (0.998–1.001)	1.000 (0.997–1.001)
Mediation proportion; % (95% CI)		0.0 (-0.7–1.5)	0.1 (-0.2–0.6)
Never or healthy drinking			
Total effect; TR (95% CI)	1.00 (ref)	0.86 (0.83–0.88)	0.64 (0.61–0.67)
Natural direct effect; TR (95% CI)		0.86 (0.83–0.88)	0.64 (0.61–0.67)
Natural indirect effect; TR (95% CI)		1.000 (1.000–1.001)	1.000 (1.000–1.001)
Mediation proportion; % (95% CI)		0.0 (-0.5–0.2)	0.0 (-0.2–0.1)
Healthy physical activity			
Total effect; TR (95% CI)	1.00 (ref)	0.84 (0.82–0.87)	0.60 (0.57–0.62)
Natural direct effect; TR (95% CI)		0.86 (0.83–0.88)	0.64 (0.61–0.67)
Natural indirect effect; TR (95% CI)		0.984 (0.980–0.987)	0.939 (0.929–0.950)
Mediation proportion; % (95% CI)		8.9 (6.8–11.9)	9.7 (7.6–11.7)
Healthy diet			
Total effect; TR (95% CI)	1.00 (ref)	0.86 (0.83–0.88)	0.63 (0.61–0.66)
Natural direct effect; TR (95% CI)		0.86 (0.83–0.88)	0.64 (0.61–0.67)
Natural indirect effect; TR (95% CI)		0.998 (0.997–1.000)	0.997 (0.995–0.999)
Mediation proportion; % (95% CI)		1.3 (0.3–2.1)	0.5 (0.2–0.9)

[a] Natural direct effect and natural indirect effect estimated the effect of frailty status on overall survival that did not or did act through the mediator (i.e., each lifestyle factor), respectively. The mediation proportion estimated the percentage of the effect of frailty status, on the log(TR) scale, that acted through the mediator (i.e., each lifestyle factor). The model was adjusted for sex, age, education, marital status, occupation prior to the age of 60 years, pension systems, residence, co-residence, and other healthy lifestyle factors.

Abbreviations: CI=confidence interval, TR=time ratio.

Supplementary Figure 1. Flow chart of participants selection for main analyses

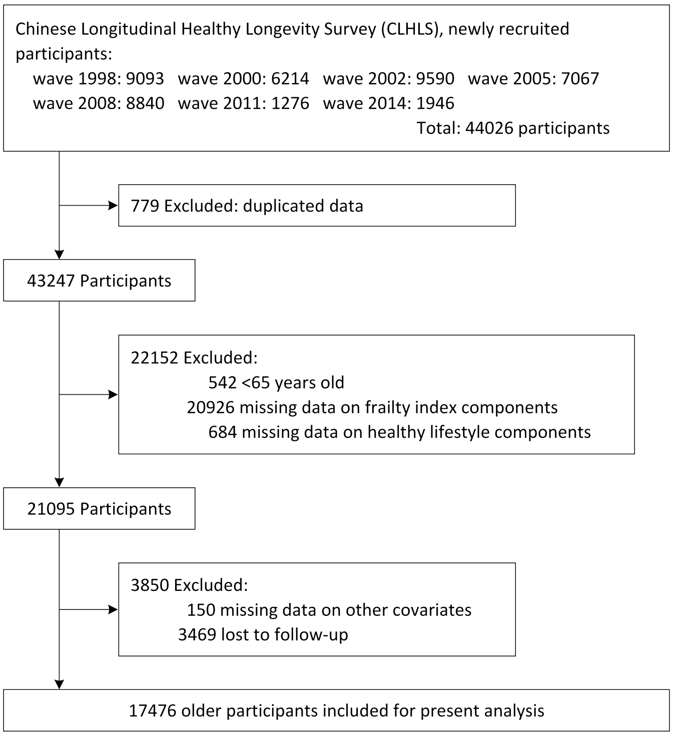

Supplementary Figure 2. Associations between frailty index, weighted healthy lifestyle score, and overall survival

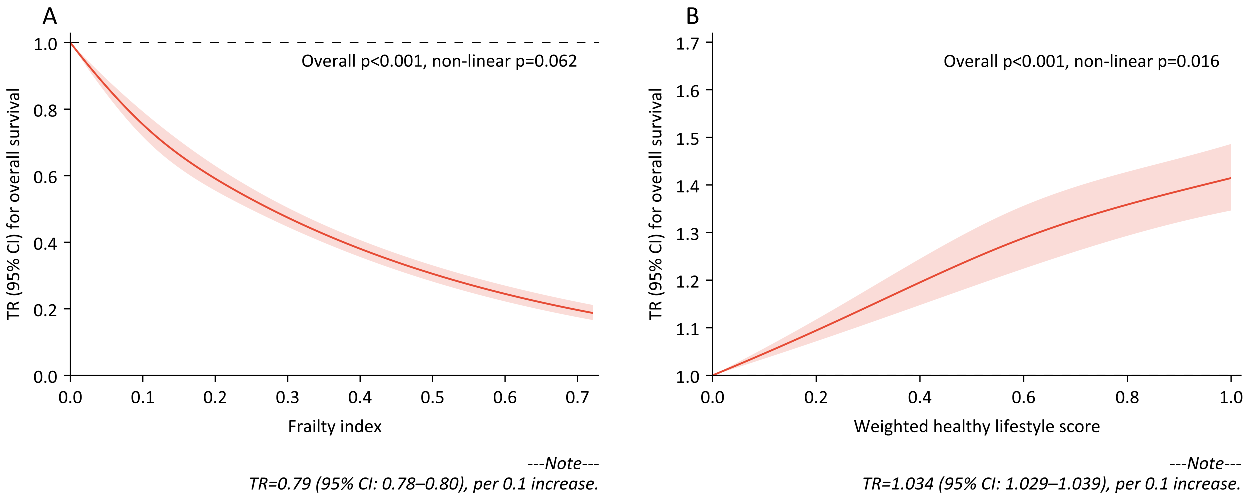

Note:

(A) The red solid line represents TRs for overall survival associated with the frailty index, while the shaded area represents the corresponding 95% CIs. The frailty index was modeled using a restricted cubic spline with three knots at the 10th, 50th, and 90th percentiles, and TRs (95% CIs) were derived from the accelerated failure model with adjustment for sex, age, education, marital status, occupation prior to the age of 60 years, pension systems, residence, and co-residence.

(B) The red solid line represents TRs for overall survival associated with the weighted healthy lifestyle score, while the shaded area represents the corresponding 95% CIs. The weighted healthy lifestyle score was modeled using a restricted cubic spline with three knots at the 10th, 50th, and 90th percentiles, and TRs (95% CIs) were derived from the accelerated failure model with adjustment for sex, age, education, marital status, occupation prior to the age of 60 years, pension systems, residence, co-residence, and frailty status.

Abbreviations: CI=confidence interval, TR=time ratio.

Supplementary Figure 3. Correlations between frailty index and weighted healthy lifestyle score

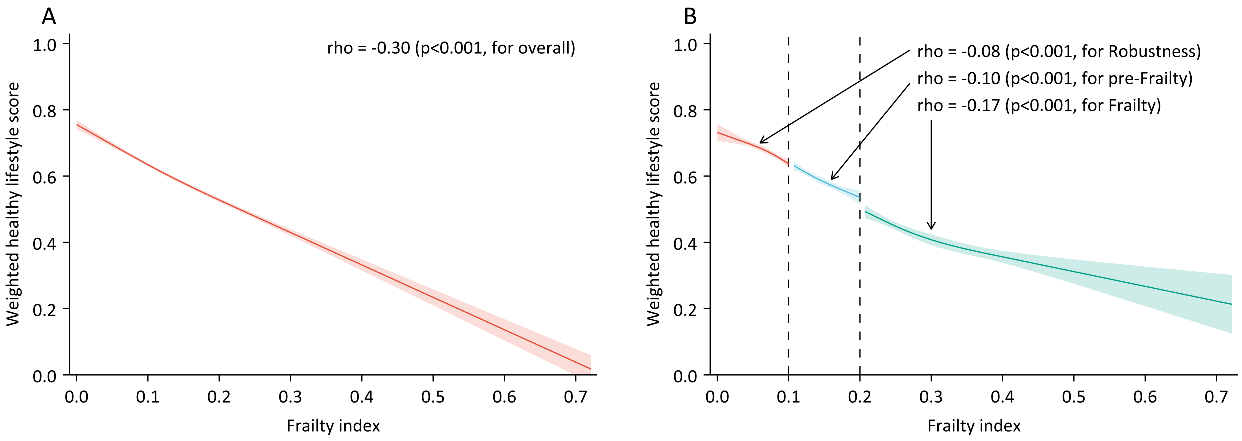

Note:

The correlation was assessed using the Spearman method, and the curve was generated by a linear regression model with a restricted cubic spline with three knots at the 10th, 50th, and 90th percentiles. The correlation between the frailty index and the simple healthy lifestyle score was found to be comparable (data not shown).

(A) For overall participants.

(B) For participants with different levels of frailty status.

Supplementary Figure 4. Associations of lifestyles with overall survival by frailty status: subgroup analysis

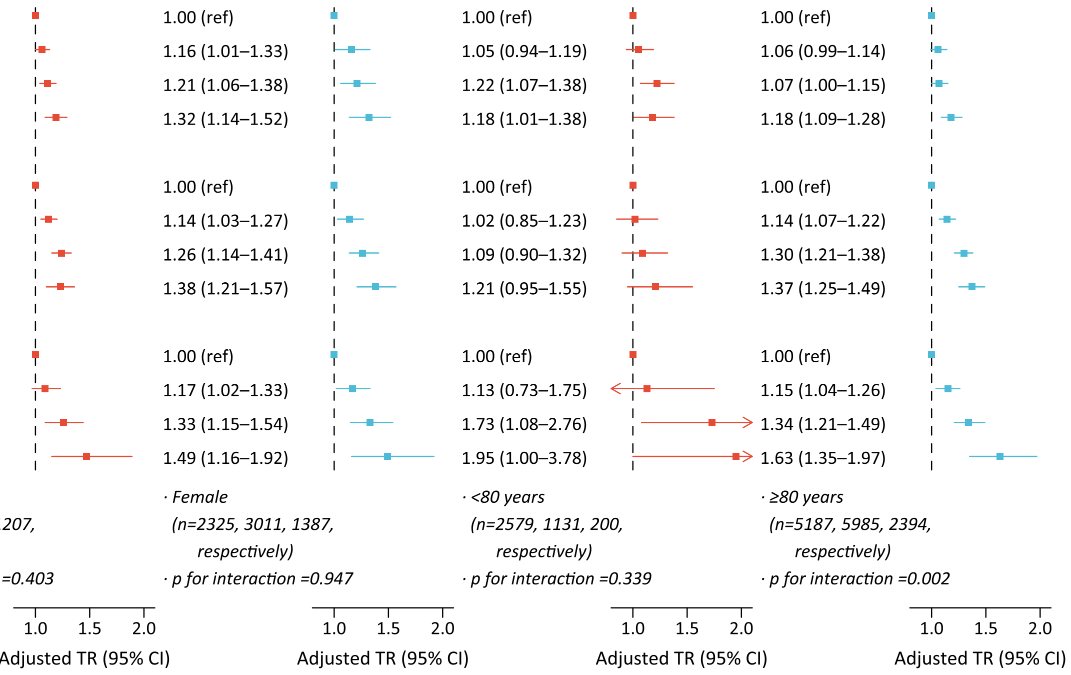

Frailty status	No. of healthy lifestyle factors	Adjusted TR (95% CI)	Adjusted TR (95% CI)	Adjusted TR (95% CI)	Adjusted TR (95% CI)
Robustness	0 or 1	1.00 (ref)	1.00 (ref)	1.00 (ref)	1.00 (ref)
	2	1.06 (1.00–1.13)	1.16 (1.01–1.33)	1.05 (0.94–1.19)	1.06 (0.99–1.14)
	3	1.11 (1.04–1.19)	1.21 (1.06–1.38)	1.22 (1.07–1.38)	1.07 (1.00–1.15)
	4	1.19 (1.09–1.29)	1.32 (1.14–1.52)	1.18 (1.01–1.38)	1.18 (1.09–1.28)
pre-Frailty	0 or 1	1.00 (ref)	1.00 (ref)	1.00 (ref)	1.00 (ref)
	2	1.12 (1.05–1.20)	1.14 (1.03–1.27)	1.02 (0.85–1.23)	1.14 (1.07–1.22)
	3	1.24 (1.15–1.33)	1.26 (1.14–1.41)	1.09 (0.90–1.32)	1.30 (1.21–1.38)
	4	1.23 (1.10–1.36)	1.38 (1.21–1.57)	1.21 (0.95–1.55)	1.37 (1.25–1.49)
Frailty	0 or 1	1.00 (ref)	1.00 (ref)	1.00 (ref)	1.00 (ref)
	2	1.09 (0.97–1.23)	1.17 (1.02–1.33)	1.13 (0.73–1.75)	1.15 (1.04–1.26)
	3	1.26 (1.09–1.44)	1.33 (1.15–1.54)	1.73 (1.08–2.76)	1.34 (1.21–1.49)
	4	1.47 (1.15–1.89)	1.49 (1.16–1.92)	1.95 (1.00–3.78)	1.63 (1.35–1.97)
		· *Male (n=5441, 4105, 1207, respectively)* · *p for interaction =0.403*	· *Female (n=2325, 3011, 1387, respectively)* · *p for interaction =0.947*	· *<80 years (n=2579, 1131, 200, respectively)* · *p for interaction =0.339*	· *≥80 years (n=5187, 5985, 2394, respectively)* · *p for interaction =0.002*

Note:
Across the 12 subgroups shown, all p-values for trend were <0.050, with the exception of one subgroup (i.e., pre-frail participants under 80 years, p=0.105).
For the adjustment of covariates, please consult the corresponding sensitivity analyses for the mediation analysis.
Abbreviations: CI=confidence interval, TR=time ratio.

Supplementary Figure 5. Associations of lifestyles with overall survival by frailty status: reverse causation, censoring losses at different time points, without comorbidities, and multiple imputation

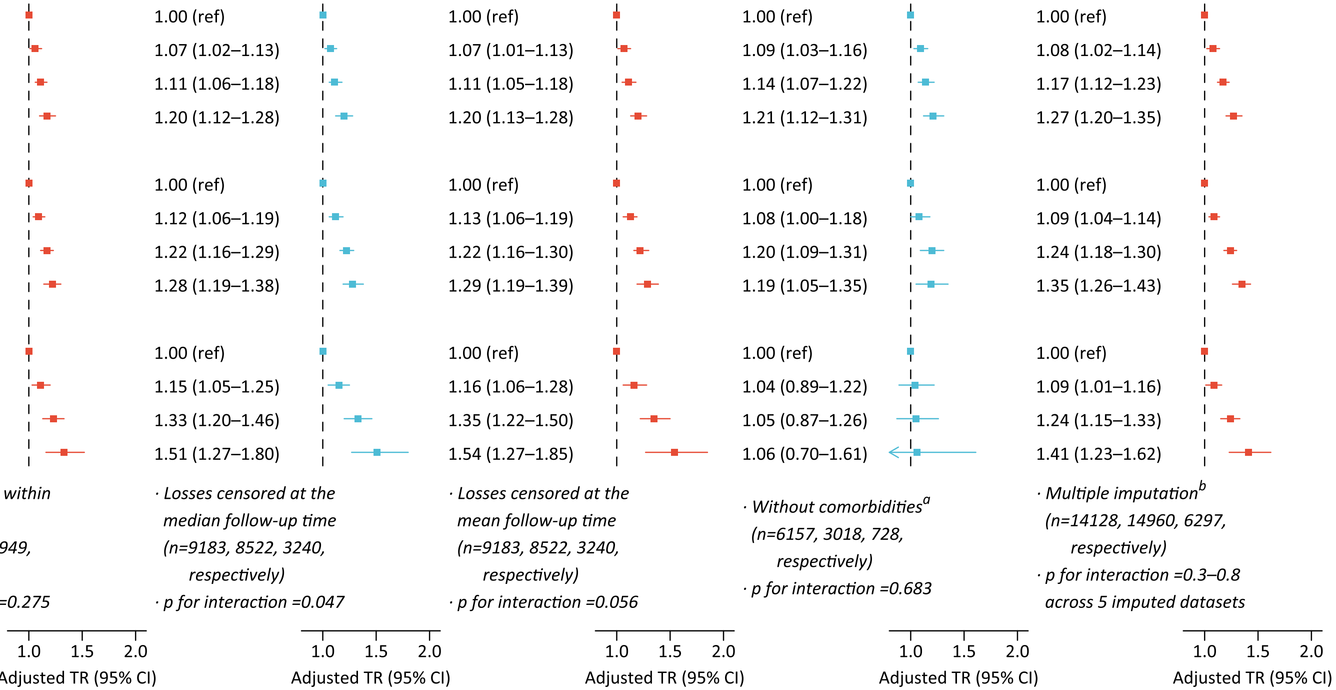

Frailty status	No. of healthy lifestyle factors	Adjusted TR (95% CI)	Adjusted TR (95% CI)	Adjusted TR (95% CI)	Adjusted TR (95% CI)	Adjusted TR (95% CI)
Robustness	0 or 1	1.00 (ref)	1.00 (ref)	1.00 (ref)	1.00 (ref)	1.00 (ref)
	2	1.06 (1.01–1.12)	1.07 (1.02–1.13)	1.07 (1.01–1.13)	1.09 (1.03–1.16)	1.08 (1.02–1.14)
	3	1.11 (1.06–1.17)	1.11 (1.06–1.18)	1.11 (1.05–1.18)	1.14 (1.07–1.22)	1.17 (1.12–1.23)
	4	1.17 (1.10–1.25)	1.20 (1.12–1.28)	1.20 (1.13–1.28)	1.21 (1.12–1.31)	1.27 (1.20–1.35)
pre-Frailty	0 or 1	1.00 (ref)	1.00 (ref)	1.00 (ref)	1.00 (ref)	1.00 (ref)
	2	1.09 (1.04–1.15)	1.12 (1.06–1.19)	1.13 (1.06–1.19)	1.08 (1.00–1.18)	1.09 (1.04–1.14)
	3	1.17 (1.11–1.23)	1.22 (1.16–1.29)	1.22 (1.16–1.30)	1.20 (1.09–1.31)	1.24 (1.18–1.30)
	4	1.22 (1.14–1.30)	1.28 (1.19–1.38)	1.29 (1.19–1.39)	1.19 (1.05–1.35)	1.35 (1.26–1.43)
Frailty	0 or 1	1.00 (ref)	1.00 (ref)	1.00 (ref)	1.00 (ref)	1.00 (ref)
	2	1.11 (1.03–1.20)	1.15 (1.05–1.25)	1.16 (1.06–1.28)	1.04 (0.89–1.22)	1.09 (1.01–1.16)
	3	1.23 (1.13–1.33)	1.33 (1.20–1.46)	1.35 (1.22–1.50)	1.05 (0.87–1.26)	1.24 (1.15–1.33)
	4	1.33 (1.16–1.52)	1.51 (1.27–1.80)	1.54 (1.27–1.85)	1.06 (0.70–1.61)	1.41 (1.23–1.62)
		· Excluding deaths within the first year (n=7255, 6222, 1949, respectively) *· p for interaction =0.275*	*· Losses censored at the median follow-up time (n=9183, 8522, 3240, respectively)* *· p for interaction =0.047*	*· Losses censored at the mean follow-up time (n=9183, 8522, 3240, respectively)* *· p for interaction =0.056*	*· Without comorbidities[a] (n=6157, 3018, 728, respectively)* *· p for interaction =0.683*	*· Multiple imputation[b] (n=14128, 14960, 6297, respectively)* *· p for interaction =0.3–0.8 across 5 imputed datasets*

Note:
Across the 12 subgroups shown, all p-values for trend were <0.001, with the exception of one subgroup (i.e., frail participants without comorbidities, p=0.610).
[a] If participants were free of all comorbidities listed in Table 1, they were classified as "without comorbidities".
[b] Multiple imputation was performed by chained equations to create five datasets, and the model estimates (TR with 95% CI) for each were combined using Rubin`s rules. For detailed information regarding multiple imputation, please refer to Supplementary Method 3.
For the adjustment of covariates, please consult the corresponding sensitivity analyses for the mediation analysis.
Abbreviations: CI=confidence interval, TR=time ratio.

Supplementary Figure 6. Associations of lifestyles with overall survival by frailty status: weighted healthy lifestyle score, cause-specific survival, SOF index, and 23-item frailty index

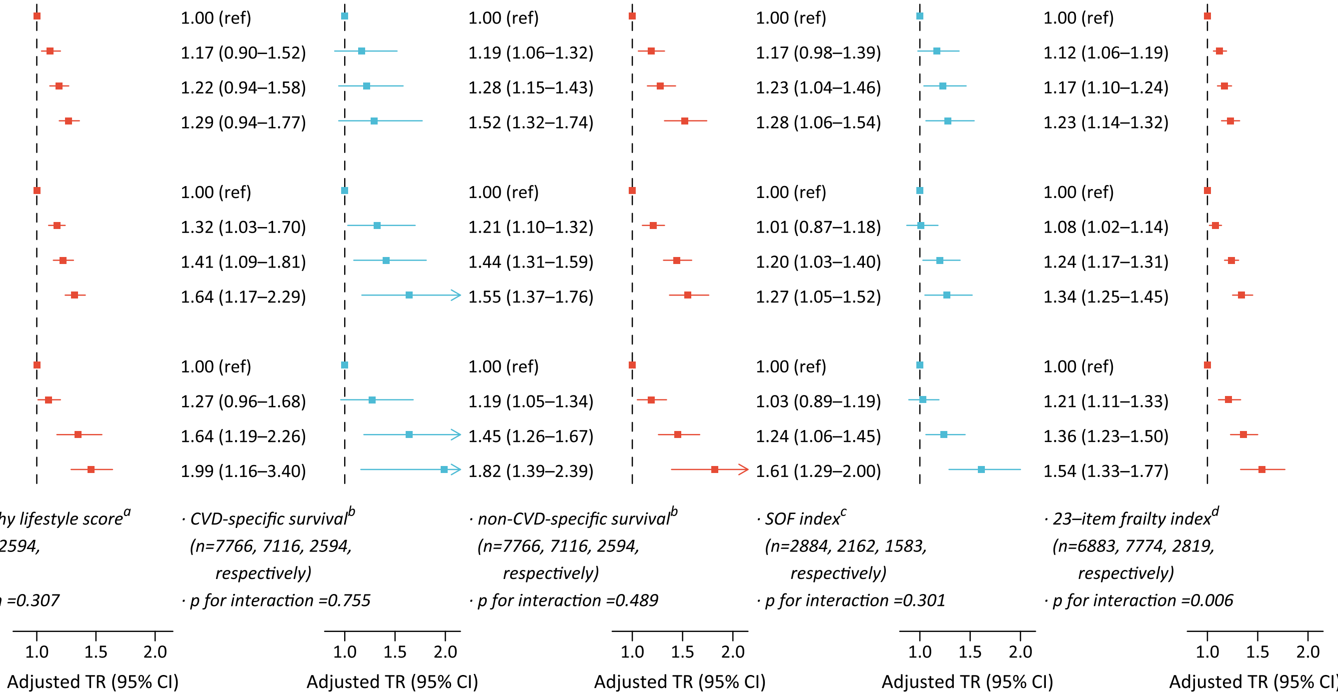

Frailty status	No. of healthy lifestyle factors	Adjusted TR (95% CI)	Adjusted TR (95% CI)	Adjusted TR (95% CI)	Adjusted TR (95% CI)	Adjusted TR (95% CI)
Robustness	0 or 1	1.00 (ref)	1.00 (ref)	1.00 (ref)	1.00 (ref)	1.00 (ref)
	2	1.11 (1.04–1.20)	1.17 (0.90–1.52)	1.19 (1.06–1.32)	1.17 (0.98–1.39)	1.12 (1.06–1.19)
	3	1.19 (1.11–1.27)	1.22 (0.94–1.58)	1.28 (1.15–1.43)	1.23 (1.04–1.46)	1.17 (1.10–1.24)
	4	1.27 (1.19–1.36)	1.29 (0.94–1.77)	1.52 (1.32–1.74)	1.28 (1.06–1.54)	1.23 (1.14–1.32)
pre-Frailty	0 or 1	1.00 (ref)	1.00 (ref)	1.00 (ref)	1.00 (ref)	1.00 (ref)
	2	1.17 (1.10–1.24)	1.32 (1.03–1.70)	1.21 (1.10–1.32)	1.01 (0.87–1.18)	1.08 (1.02–1.14)
	3	1.22 (1.14–1.31)	1.41 (1.09–1.81)	1.44 (1.31–1.59)	1.20 (1.03–1.40)	1.24 (1.17–1.31)
	4	1.32 (1.24–1.41)	1.64 (1.17–2.29)	1.55 (1.37–1.76)	1.27 (1.05–1.52)	1.34 (1.25–1.45)
Frailty	0 or 1	1.00 (ref)	1.00 (ref)	1.00 (ref)	1.00 (ref)	1.00 (ref)
	2	1.10 (1.01–1.20)	1.27 (0.96–1.68)	1.19 (1.05–1.34)	1.03 (0.89–1.19)	1.21 (1.11–1.33)
	3	1.35 (1.17–1.55)	1.64 (1.19–2.26)	1.45 (1.26–1.67)	1.24 (1.06–1.45)	1.36 (1.23–1.50)
	4	1.46 (1.29–1.64)	1.99 (1.16–3.40)	1.82 (1.39–2.39)	1.61 (1.29–2.00)	1.54 (1.33–1.77)
		· *Weighted healthy lifestyle score*[a] *(n=7766, 7116, 2594, respectively)* · *p for interaction =0.307*	· *CVD-specific survival*[b] *(n=7766, 7116, 2594, respectively)* · *p for interaction =0.755*	· *non-CVD-specific survival*[b] *(n=7766, 7116, 2594, respectively)* · *p for interaction =0.489*	· *SOF index*[c] *(n=2884, 2162, 1583, respectively)* · *p for interaction =0.301*	· *23–item frailty index*[d] *(n=6883, 7774, 2819, respectively)* · *p for interaction =0.006*

Note:

Across the 15 subgroups shown, all p-values for trend were <0.050, with the exception of one subgroup (i.e., robust participants in assessing CVD-specific survival, p=0.117).

[a] The weighted healthy lifestyle score was grouped by quartiles: quartile 1 = no or one healthy lifestyle factor, quartile 2 = two healthy lifestyle factors, quartile 3 = three healthy lifestyle factors, and quartile 4 = four healthy lifestyle factors.

[b] For detailed information regarding cause-specific survival, please refer to Supplementary Method 3.

[c] Frailty status was defined by the study of osteoporotic fractures index. For detailed information, please refer to Supplementary Method 3.

[d] Frailty status was defined by the 23-item frailty index. For detailed information, please refer to Supplementary Method 3.

For the adjustment of covariates, please consult the corresponding sensitivity analyses for the mediation analysis.

Abbreviations: CI=confidence interval, CVD= cardiovascular disease, SOF=study of osteoporotic fractures, TR=time ratio.

Supplementary Figure 7. Joint associations of frailty status and lifestyles with overall survival: subgroup analysis

Frailty status	No. of healthy lifestyle factors	Adjusted TR (95% CI)	Adjusted TR (95% CI)	p for interaction
		Sex: male	Sex: female	
Robustness	4	1.00 (ref)	1.00 (ref)	
	3	0.94 (0.86–1.02)	0.92 (0.83–1.01)	
	2	0.89 (0.82–0.97)	0.87 (0.78–0.97)	
	0 or 1	0.83 (0.76–0.91)	0.74 (0.64–0.87)	
pre-Frailty	4	0.81 (0.72–0.90)	0.89 (0.79–0.99)	
	3	0.82 (0.75–0.89)	0.82 (0.75–0.90)	0.608
	2	0.74 (0.68–0.81)	0.74 (0.68–0.82)	
	0 or 1	0.66 (0.60–0.72)	0.65 (0.57–0.74)	
Frailty	4	0.67 (0.54–0.83)	0.66 (0.54–0.82)	
	3	0.58 (0.51–0.66)	0.60 (0.54–0.68)	
	2	0.52 (0.46–0.58)	0.53 (0.48–0.59)	
	0 or 1	0.47 (0.43–0.53)	0.46 (0.40–0.52)	
		Age: <80 years	Age: ≥80 years	
Robustness	4	1.00 (ref)	1.00 (ref)	
	3	1.03 (0.90–1.19)	0.91 (0.84–0.98)	
	2	0.90 (0.78–1.03)	0.89 (0.82–0.96)	
	0 or 1	0.85 (0.72–1.00)	0.83 (0.76–0.90)	
pre-Frailty	4	0.85 (0.69–1.04)	0.80 (0.73–0.88)	
	3	0.76 (0.65–0.89)	0.76 (0.70–0.82)	0.515
	2	0.72 (0.61–0.84)	0.67 (0.62–0.72)	
	0 or 1	0.70 (0.58–0.84)	0.59 (0.54–0.64)	
Frailty	4	0.69 (0.46–1.05)	0.59 (0.50–0.70)	
	3	0.63 (0.47–0.84)	0.49 (0.45–0.54)	
	2	0.43 (0.33–0.56)	0.42 (0.39–0.46)	
	0 or 1	0.39 (0.31–0.50)	0.38 (0.34–0.41)	

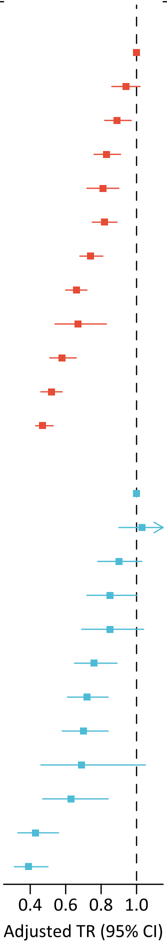

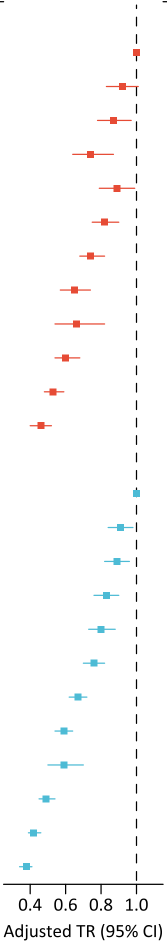

Note:
Across the four subgroups shown, all p-values for trend were <0.001.
For the adjustment of covariates, please consult the corresponding sensitivity analyses for the mediation analysis.
Abbreviations: CI=confidence interval, TR=time ratio.

Supplementary Figure 8. Joint associations of frailty status and lifestyles with overall survival: reverse causation, censoring losses at different time points, without comorbidities, and multiple imputation

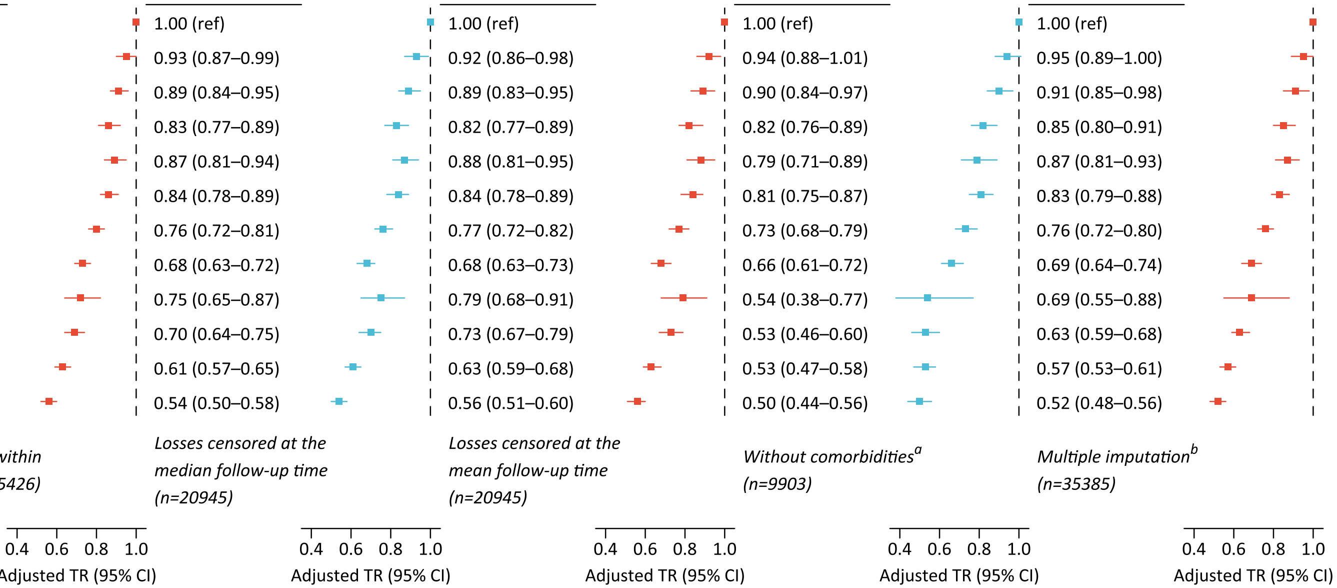

Frailty status	No. of healthy lifestyle factors	Adjusted TR (95% CI) — *Excluding deaths within the first year (n=15426)*	Adjusted TR (95% CI) — *Losses censored at the median follow-up time (n=20945)*	Adjusted TR (95% CI) — *Losses censored at the mean follow-up time (n=20945)*	Adjusted TR (95% CI) — *Without comorbidities[a] (n=9903)*	Adjusted TR (95% CI) — *Multiple imputation[b] (n=35385)*
Robustness	4	1.00 (ref)	1.00 (ref)	1.00 (ref)	1.00 (ref)	1.00 (ref)
	3	0.95 (0.90–1.00)	0.93 (0.87–0.99)	0.92 (0.86–0.98)	0.94 (0.88–1.01)	0.95 (0.89–1.00)
	2	0.91 (0.87–0.96)	0.89 (0.84–0.95)	0.89 (0.83–0.95)	0.90 (0.84–0.97)	0.91 (0.85–0.98)
	0 or 1	0.86 (0.81–0.92)	0.83 (0.77–0.89)	0.82 (0.77–0.89)	0.82 (0.76–0.89)	0.85 (0.80–0.91)
pre-Frailty	4	0.89 (0.84–0.95)	0.87 (0.81–0.94)	0.88 (0.81–0.95)	0.79 (0.71–0.89)	0.87 (0.81–0.93)
	3	0.86 (0.82–0.91)	0.84 (0.78–0.89)	0.84 (0.78–0.89)	0.81 (0.75–0.87)	0.83 (0.79–0.88)
	2	0.80 (0.76–0.84)	0.76 (0.72–0.81)	0.77 (0.72–0.82)	0.73 (0.68–0.79)	0.76 (0.72–0.80)
	0 or 1	0.73 (0.69–0.77)	0.68 (0.63–0.72)	0.68 (0.63–0.73)	0.66 (0.61–0.72)	0.69 (0.64–0.74)
Frailty	4	0.72 (0.64–0.82)	0.75 (0.65–0.87)	0.79 (0.68–0.91)	0.54 (0.38–0.77)	0.69 (0.55–0.88)
	3	0.69 (0.64–0.74)	0.70 (0.64–0.75)	0.73 (0.67–0.79)	0.53 (0.46–0.60)	0.63 (0.59–0.68)
	2	0.63 (0.59–0.67)	0.61 (0.57–0.65)	0.63 (0.59–0.68)	0.53 (0.47–0.58)	0.57 (0.53–0.61)
	0 or 1	0.56 (0.52–0.60)	0.54 (0.50–0.58)	0.56 (0.51–0.60)	0.50 (0.44–0.56)	0.52 (0.48–0.56)

Note:
Across the four types of sensitivity analyses shown, all p-values for trend were <0.001.
[a] If participants were free of all comorbidities listed in Table 1, they were classified as "without comorbidities".
[b] Multiple imputation was performed by chained equations to create five datasets, and the model estimates (TR with 95% CI) for each were combined using Rubin`s rules. For detailed information regarding multiple imputation, please refer to Supplementary Method 3.
For the adjustment of covariates, please consult the corresponding sensitivity analyses for the mediation analysis.
Abbreviations: CI=confidence interval, TR=time ratio.

Supplementary Figure 9. Joint associations of frailty status and lifestyles with overall survival: weighted healthy lifestyle score, cause-specific survival, SOF index, and 23-item frailty index

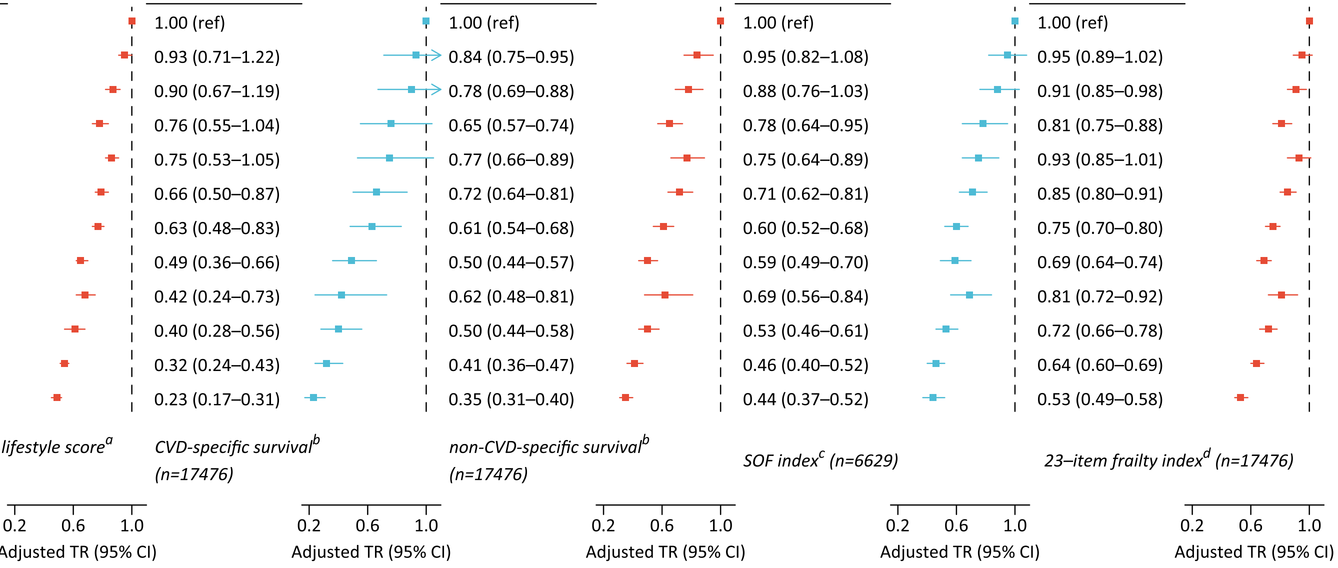

Frailty status	No. of healthy lifestyle factors	Adjusted TR (95% CI) — Weighted healthy lifestyle score[a] (n=17476)	Adjusted TR (95% CI) — CVD-specific survival[b] (n=17476)	Adjusted TR (95% CI) — non-CVD-specific survival[b] (n=17476)	Adjusted TR (95% CI) — SOF index[c] (n=6629)	Adjusted TR (95% CI) — 23–item frailty index[d] (n=17476)
Robustness	4	1.00 (ref)	1.00 (ref)	1.00 (ref)	1.00 (ref)	1.00 (ref)
	3	0.95 (0.91–1.00)	0.93 (0.71–1.22)	0.84 (0.75–0.95)	0.95 (0.82–1.08)	0.95 (0.89–1.02)
	2	0.87 (0.82–0.92)	0.90 (0.67–1.19)	0.78 (0.69–0.88)	0.88 (0.76–1.03)	0.91 (0.85–0.98)
	0 or 1	0.78 (0.73–0.84)	0.76 (0.55–1.04)	0.65 (0.57–0.74)	0.78 (0.64–0.95)	0.81 (0.75–0.88)
pre-Frailty	4	0.86 (0.82–0.91)	0.75 (0.53–1.05)	0.77 (0.66–0.89)	0.75 (0.64–0.89)	0.93 (0.85–1.01)
	3	0.79 (0.75–0.84)	0.66 (0.50–0.87)	0.72 (0.64–0.81)	0.71 (0.62–0.81)	0.85 (0.80–0.91)
	2	0.77 (0.73–0.81)	0.63 (0.48–0.83)	0.61 (0.54–0.68)	0.60 (0.52–0.68)	0.75 (0.70–0.80)
	0 or 1	0.65 (0.62–0.70)	0.49 (0.36–0.66)	0.50 (0.44–0.57)	0.59 (0.49–0.70)	0.69 (0.64–0.74)
Frailty	4	0.68 (0.62–0.75)	0.42 (0.24–0.73)	0.62 (0.48–0.81)	0.69 (0.56–0.84)	0.81 (0.72–0.92)
	3	0.61 (0.54–0.68)	0.40 (0.28–0.56)	0.50 (0.44–0.58)	0.53 (0.46–0.61)	0.72 (0.66–0.78)
	2	0.54 (0.51–0.57)	0.32 (0.24–0.43)	0.41 (0.36–0.47)	0.46 (0.40–0.52)	0.64 (0.60–0.69)
	0 or 1	0.49 (0.45–0.52)	0.23 (0.17–0.31)	0.35 (0.31–0.40)	0.44 (0.37–0.52)	0.53 (0.49–0.58)

Note:

Across the five types of sensitivity analyses shown, all p-values for trend were <0.001.

[a] The weighted healthy lifestyle score was grouped by quartiles: quartile 1 = no or one healthy lifestyle factor, quartile 2 = two healthy lifestyle factors, quartile 3 = three healthy lifestyle factors, and quartile 4 = four healthy lifestyle factors.

[b] For detailed information regarding cause-specific survival, please refer to Supplementary Method 3.

[c] Frailty status was defined by the study of osteoporotic fractures index. For detailed information, please refer to Supplementary Method 3.

[d] Frailty status was defined by the 23-item frailty index. For detailed information, please refer to Supplementary Method 3.

For the adjustment of covariates, please consult the corresponding sensitivity analyses for the mediation analysis.

Abbreviations: CI=confidence interval, CVD= cardiovascular disease, SOF=study of osteoporotic fractures, TR=time ratio.

Supplementary Figure 10. Estimated remaining life expectancy at the age of 65 years

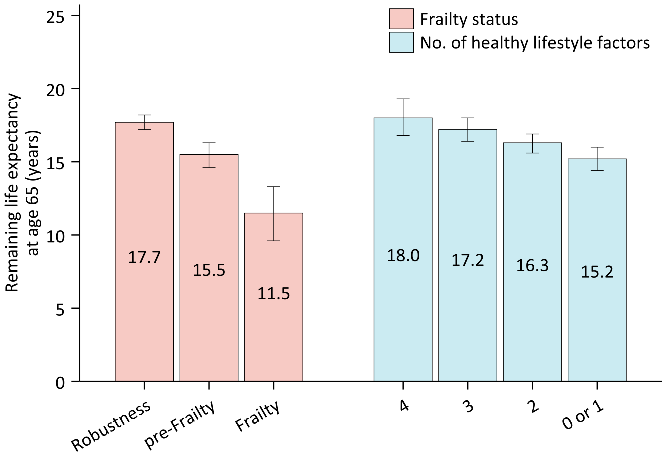

Supplementary Figure 11. Attribution of the causes of death

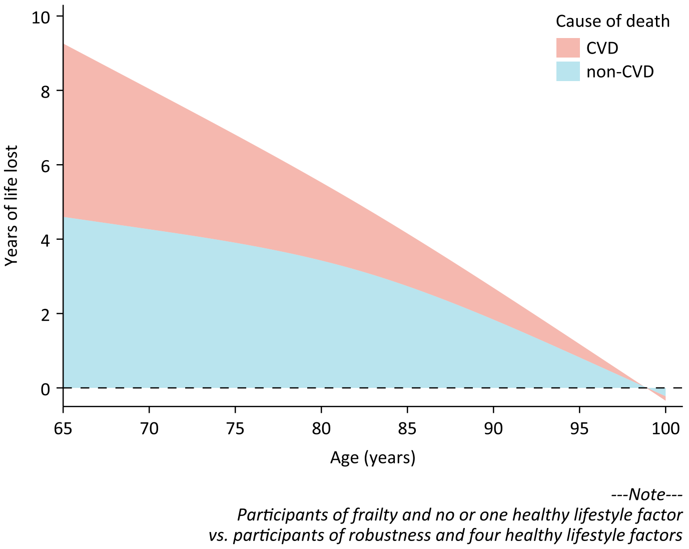

Note:
When comparing participants of frailty and no or one healthy lifestyle factor to those of robustness and four healthy lifestyle factors, the weighted average of years of life lost after the age of 65 was found to be 4.0 years for the former group. The loss was due to increased deaths from CVD (1.7 years, accounting for 42.5% of the total years lost) and non-CVD causes (2.3 years, accounting for 57.5% of the total years lost).
Abbreviations: CVD= cardiovascular disease.

Supplementary file: STROBE checklist—all the items listed below have been confirmed, and item 6(b) is not relevant to the present study

	Item No	Recommendation
**Title and abstract**	1	(*a*) Indicate the study's design with a commonly used term in the title or the abstract
		(*b*) Provide in the abstract an informative and balanced summary of what was done and what was found
**Introduction**		
Background/rationale	2	Explain the scientific background and rationale for the investigation being reported
Objectives	3	State specific objectives, including any prespecified hypotheses
**Methods**		
Study design	4	Present key elements of study design early in the paper
Setting	5	Describe the setting, locations, and relevant dates, including periods of recruitment, exposure, follow-up, and data collection
Participants	6	(*a*) Give the eligibility criteria, and the sources and methods of selection of participants. Describe methods of follow-up
		(*b*) For matched studies, give matching criteria and number of exposed and unexposed
Variables	7	Clearly define all outcomes, exposures, predictors, potential confounders, and effect modifiers. Give diagnostic criteria, if applicable
Data sources/ measurement	8*	For each variable of interest, give sources of data and details of methods of assessment (measurement). Describe comparability of assessment methods if there is more than one group
Bias	9	Describe any efforts to address potential sources of bias
Study size	10	Explain how the study size was arrived at
Quantitative variables	11	Explain how quantitative variables were handled in the analyses. If applicable, describe which groupings were chosen and why
Statistical methods	12	(*a*) Describe all statistical methods, including those used to control for confounding
		(*b*) Describe any methods used to examine subgroups and interactions
		(*c*) Explain how missing data were addressed
		(*d*) If applicable, explain how loss to follow-up was addressed
		(*e*) Describe any sensitivity analyses
**Results**		
Participants	13*	(a) Report numbers of individuals at each stage of study—eg numbers potentially eligible, examined for eligibility, confirmed eligible, included in the study, completing follow-up, and analysed
		(b) Give reasons for non-participation at each stage
		(c) Consider use of a flow diagram
Descriptive data	14*	(a) Give characteristics of study participants (eg demographic, clinical, social) and information on exposures and potential confounders
		(b) Indicate number of participants with missing data for each variable of interest
		(c) Summarise follow-up time (eg, average and total amount)
Outcome data	15*	Report numbers of outcome events or summary measures over time
Main results	16	(*a*) Give unadjusted estimates and, if applicable, confounder-adjusted estimates and their precision (eg, 95% confidence interval). Make clear which confounders were adjusted for and why they were included
		(*b*) Report category boundaries when continuous variables were categorized
		(*c*) If relevant, consider translating estimates of relative risk into absolute risk for a meaningful time period
Other analyses	17	Report other analyses done—eg analyses of subgroups and interactions, and sensitivity analyses
**Discussion**		
Key results	18	Summarise key results with reference to study objectives
Limitations	19	Discuss limitations of the study, taking into account sources of potential bias or imprecision. Discuss both direction and magnitude of any potential bias

	Item No	Recommendation
Interpretation	20	Give a cautious overall interpretation of results considering objectives, limitations, multiplicity of analyses, results from similar studies, and other relevant evidence
Generalisability	21	Discuss the generalisability (external validity) of the study results
**Other information**		
Funding	22	Give the source of funding and the role of the funders for the present study and, if applicable, for the original study on which the present article is based

*Give information separately for exposed and unexposed groups.

Note: An Explanation and Elaboration article discusses each checklist item and gives methodological background and published examples of transparent reporting. The STROBE checklist is best used in conjunction with this article (freely available on the Web sites of PLoS Medicine at http://www.plosmedicine.org/, Annals of Internal Medicine at http://www.annals.org/, and Epidemiology at http://www.epidem.com/). Information on the STROBE Initiative is available at http://www.strobe-statement.org.
